# A Stereocontrolled Protocol to Highly Functionalized Fluorinated Scaffolds through a Fluoride Opening of Oxiranes

**DOI:** 10.3390/molecules21111493

**Published:** 2016-11-17

**Authors:** Attila Márió Remete, Melinda Nonn, Santos Fustero, Ferenc Fülöp, Loránd Kiss

**Affiliations:** 1 Institute of Pharmaceutical Chemistry, University of Szeged, Eötvös u. 6, H-6720 Szeged, Hungary; remete.attila@pharm.u-szeged.hu (A.M.R.); fulop@pharm.u-szeged.hu (F.F.); 2 MTA-SZTE Stereochemistry Research Group, Hungarian Academy of Sciences, Eötvös u. 6, H-6720 Szeged, Hungary; nonn.melinda@pharm.u-szeged.hu; 3 Departamento de Química Orgánica, Facultad de Farmacia, Universidad de Valencia, Av. Vicente Andrés Estellés, s/n 46100 Valencia, Spain; Santos.Fustero@uv.es

**Keywords:** oxirane, fluorination, amino acids, stereoselectivity, regioselectivity

## Abstract

A novel selective and substrate-dependent synthetic protocol has been developed towards the synthesis of various fluorine-containing, highly functionalized cycloalkane derivatives. The method involves the stereoselective epoxidation of some unsaturated cyclic β-amino acid derivatives as model compounds, followed by a regioselective fluoride opening of oxiranes under various conditions with Deoxofluor and XtalFluor-E reagents, thereby offering an insight into this new epoxide opening methodology with fluoride.

## 1. Introduction

Olefin epoxidation and the ring opening of epoxides with various nucleophiles is a widely used method for the creation of different functional groups on the skeleton of organic molecules [1,2,3,4,5,6,7].

Because of the increasing importance of fluorinated biomolecules, the introduction of one or more fluorine atoms into an organic molecule has received great interest in recent years. Besides the important fluorine-containing drugs [8,9], several fluorinated α- and acyclic β-amino acids are known as enzyme inhibitors, antitumoral agents, and antibiotics [10,11,12,13].

Taking into consideration the significance of fluorine in the structure of an organic molecule, an oxirane ring opening with fluoride is a substantially useful approach for the incorporation of a fluorine atom into a certain molecule [14,15,16,17]. However, the stereo- and regioselectivity in highly functionalized cyclic scaffolds is a major challenge, which is associated with this type of transformation. Various synthetic methods including asymmetric approaches for the oxirane ring opening with fluoride, using fluorine-containing reagents such as pyridine·HF [18,19], Et_3_N·3HF [20,21], BF_3_·OEt_2_ [22,23], HBF_4_·OEt_2_ or HBF_4_ [24,25], KHF_2_ [26], TBAF/KHF_2_, TBAF·3H_2_O/KF [27], KHF_2_/18-crown-6/chiral Jacobsen chromium complex [28,29], AgHF_2_ with Ru catalyst [30], benzoyl fluoride, base, and HFIP [31,32], have been reported. However, despite the relatively large number of synthetic opportunities listed here, these molecular entities possessing the bicyclic oxirane fused ring system are still considered complicated substrates for ring openings with fluoride. Consequently, there might be a need for the development of novel synthetic techniques in view of this transformation.

## 2. Results and Discussion

Our goal has been the development of a novel synthetic approach for the introduction of a fluorine atom into the skeleton of a highly functionalized cycloalkane framework. We designed here a two-step protocol: first, selective epoxidation; second, a selective oxirane ring opening with appropriately selected fluoride reagents.

As a consequence of their important biological potential, highly functionalized amino acid derivatives play a significant role in medicinal and organic chemistry. Some functionalized alicyclic amino acid derivatives, such as oryzoxymycin, oseltamivir, peramivir, and icofungipen, exert relevant bioactive properties [33].

There are relatively few methods in the literature [5,10,11,12,13] for the access of fluorine-containing, highly functionalized β-amino acid derivatives. Therefore, the synthetic protocol for the access of various fluorine-containing functionalized scaffolds regarding these valuable bioactive derivatives was investigated here for the first time. The synthetic concept involves the stereoselective epoxidation of some unsaturated β-amino esters (derived from bicyclic β-lactams) as selected model compounds. In the next step, the regioselective fluoride opening of the formed oxiranes with both Deoxofluor and XtalFluor-E reagents is carried out for the creation of novel, potentially bioactive, fluorine-containing β-amino acid derivatives or heterocyclic systems.

**Scheme 1:**
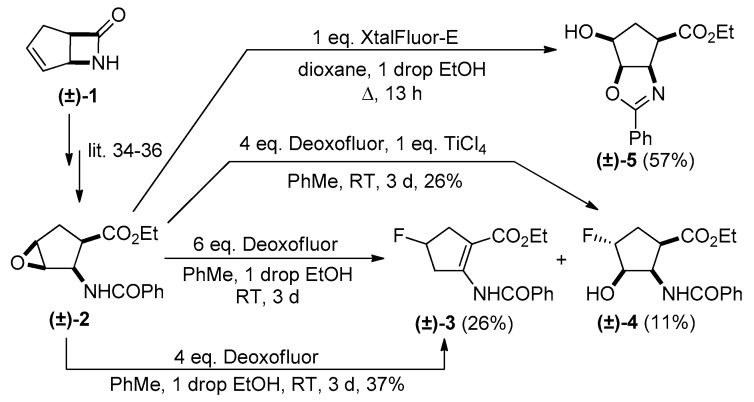
Oxirane opening of *all-cis* epoxy amino ester **(±)-2** with Deoxofluor and XtalFluor-E.

**Scheme 2:**
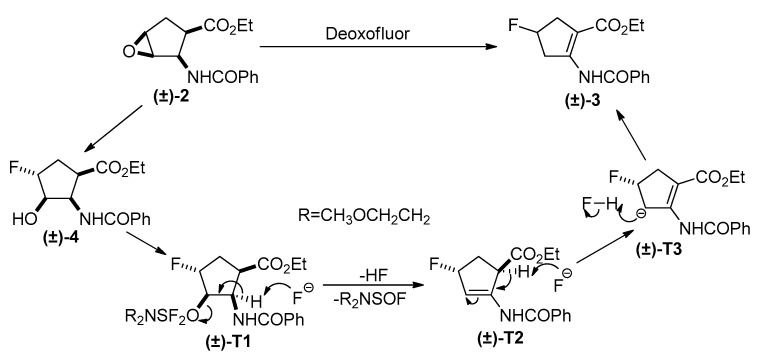
A possible route for the formation of fluorinated amino ester** (±)-3**.

First, the fluorination procedures were investigated with cyclopentane β-amino acid derivatives. For this reason, a number of experiments were performed with systematic variation in the fluorinating agent, the number of equivalents, the solvent, the temperature, the reaction time, and the usage of additives. The *all-cis* epoxy amino ester **(±)-2** (derived from bicyclic lactam **(±)-1** according to our earlier protocol) [34,35,36] was treated with XtalFluor-E [37] in dioxane at reflux (see Method D in the Materials and Methods section). The opening of the oxirane ring yielded an oxazolidine derivative **(±)-5** as the sole product through intramolecular nucleophilic cyclization by the amide *O*-atom (Scheme 1). It is noteworthy that the same reaction, when carried out in other common solvents such as PhMe, CH_2_Cl_2_, and THF, yielded no transformation; only starting epoxide could be detected after a day of reflux. Unfortunately, compound **(±)-5**, when reacted with Deoxofluor [38] under various conditions (in different solvents at room temperature, reflux, etc.), did not yield the corresponding fluorinated derivative. Next, the fluorination regent was changed from XtalFluor-E to Deoxofluor. When epoxide **(±)-2** was reacted with 6 equiv. of Deoxofluor in toluene (after some evaluation of various solvents such as THF, 1,4-dioxane, and CH_2_Cl_2_, this proved to be the most efficient) in the presence of one drop of EtOH at 20 °C for three days (see Method A in the Materials and Methods section), two products were isolated: One was an unsaturated fluorinated derivative **(±)-3** as a result of the elimination and rearrangement (see also Scheme 2). The other product was an amino ester **(±)-4** containing both fluorine and a hydroxyl group (Scheme 1). Derivatives **(±)-3** and **(±)-4** formed in a 2.3:1 ratio were separated and isolated by means of column chromatography. Although compound **(±)-2**, depending on the reagent, yielded different types of products, by reacting either with XtalFluor or Deoxofluor, the reactions with these reagents (analogously to the hydroxy–fluorine exchange; see [37] and references therein) most likely took place with different mechanisms. The exact elucidation of these mechanisms needs deeper evaluation, which is in progress in our laboratory.

The oxirane ring opening with both XtalFluor-E and Deoxofluor was studied in various solvents (e.g., THF, CH_2_Cl_2_, dioxane, and PhMe) and in the presence of certain additives activating the oxiranes. After some experimental investigations using different electrophilic additives (e.g., BF_3_·OEt_2_, NH_4_Cl, AlCl_3_, and Li salts), the ring opening of oxirane **(±)-2** was achieved with 4 equiv. of Deoxofluor in the presence of TiCl_4_ (see Method C in the Material and Methods section), furnishing compound **(±)-4** as the only product, albeit in a modest yield (26%) (Scheme 1). It is noteworthy that, in all fluorination transformations, alongside with the desired fluorine-containing products, either a significant amount of starting epoxide or a polymeric material could be detected.

**Scheme 3:**
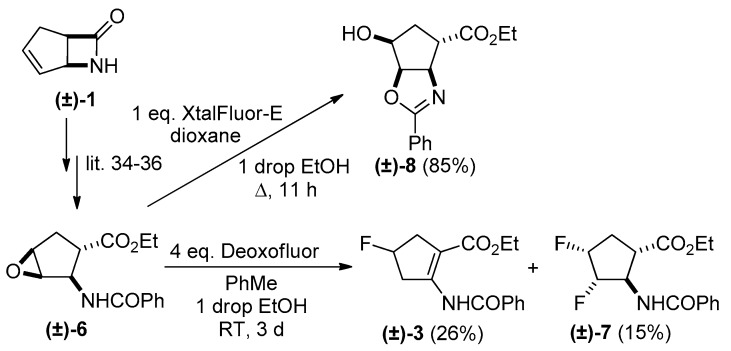
Oxirane opening of the “*trans*” epoxy amino ester **(±)-6** with Deoxofluor and XtalFluor-E.

Similar studies have been carried out with “*trans*” epoxy amino ester **(±)-6** (synthetized analogously as reported earlier [34,35,36]), in which the ester and the carbamate groups are in a relative *trans* arrangement. In the reaction with XtalFluor-E (reflux in dioxane), epoxy amino ester **(±)-6** provided a cyclized derivative **(±)-8** (see Method D in the Materials and Methods section). Deoxofluor, again, yielded two products in a 1.7:1 ratio. One is unsaturated amino ester **(±)-3**, the same product formed from *all-cis *amino esters **(±)-2** (Scheme 1), while the second is a difluorinated derivative **(±)-7** (see Method B in the Materials and Methods section) (Scheme 3).

**Scheme 4:**
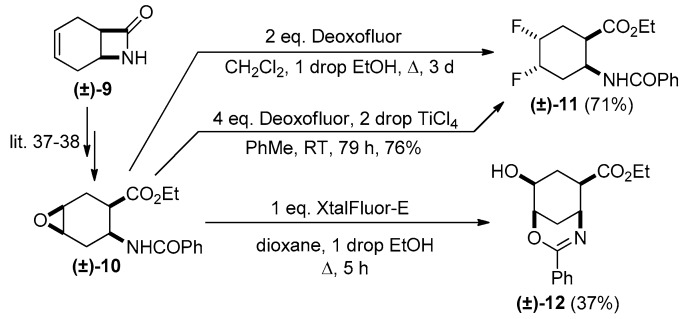
Oxirane opening of cyclohexane *all-cis* epoxy amino ester **(±)-10** with Deoxofluor and XtalFluor-E.

Our synthetic investigations were further extended for larger ring systems, namely the six-membered analogues. Thus, *all-cis* epoxy amino ester **(±)-10** (derived from bicyclic lactam **(±)-9** according to a known method) [39,40] was subjected to fluorination. When the reaction was accomplished with 2 equiv. of Deoxofluor (CH_2_Cl_2_ and reflux), difluorinated derivative **(±)-11** was obtained. Note that a similar product could not be detected in the transformation of cyclopentane derivative **(±)-2**. The yield of fluorinated amino ester **(±)-11** might be slightly increased (76%) by the addition of TiCl_4_ (Method C). Furthermore, the reaction of compound **(±)-10** with XtalFluor-E afforded oxazine derivative **(±)-12**, formed via the intramolecular ring closing (Method D) (Scheme 4).

**Scheme 5:**
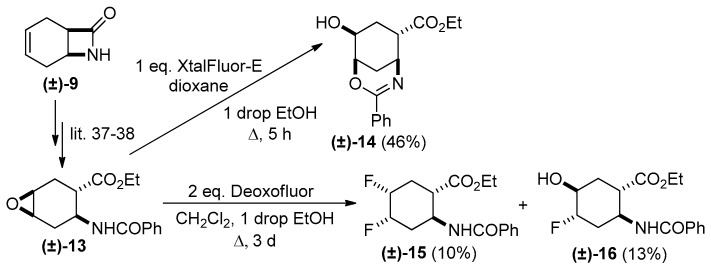
Oxirane opening of cyclohexane “*trans*” amino ester **(±)-13** with Deoxofluor and XtalFluor-E.

However, rather surprisingly, the result of the fluorination of “*trans*” epoxy amino ester isomer **(±)-13** (for similar epoxidation see also reference [39,40]) is different from that of the *all-cis* isomer **(±)-10**. The reaction of **(±)-13** with Deoxofluor resulted in difluorinated amino ester **(±)-15** and fluoroalcohol derivative **(±)-16** formed in low yields (Method B). Next, the fluorination of **(±)-13** accomplished with XtalFluor-E (reflux in dioxane) furnished oxazine derivative **(±)-14**, as a single product, in a modest yield (Method D) (Scheme 5). It is noteworthy, however, that, contrary to epoxide **(±)-2**, fluorination of **(±)-10** in the presence of TiCl_4_ yielded no identifiable product.

## 3. Materials and Methods

Epoxidations of cycloalkene β-amino esters were performed according to procedures published previously [34,39]. The ^1^H-NMR and ^13^C-NMR spectra of all new compounds are available in the Supplementary Materials.

### Experimental Procedures for the Oxirane Ring Openings

Method A: To a solution of epoxide (0.5–0.7 mmol) in anhydrous toluene (10 mL) under an Ar atmosphere, one drop of anhydrous EtOH and 50% Deoxofluor in toluene (4 equiv.) were added, and the solution was stirred at 20 °C for the time given in the schemes. The solution was then diluted with CH_2_Cl_2_ (30 mL), and the organic layer was washed with a saturated aqueous NaHCO_3_ solution (2 × 20 mL), dried (Na_2_SO_4_), and concentrated. The crude product was purified by column chromatography on silica gel (*n*-hexane/EtOAc).

Method B: To a solution of epoxide (0.5 mmol) in anhydrous CH_2_Cl_2_ (10 mL) under an Ar atmosphere, one drop of anhydrous EtOH and 50% Deoxofluor in toluene (1 equiv.) were added, and the solution was kept at reflux temperature for 2 days. Then, 50% Deoxofluor in toluene (1 equiv.) was added again, and the solution was treated as above for one more day. The solution was then diluted with CH_2_Cl_2_ (30 mL), and the organic layer was washed with saturated aqueous NaHCO_3_ solution (2 × 20 mL), dried (Na_2_SO_4_), and concentrated. The crude product was purified by column chromatography on silica gel (*n*-hexane/EtOAc).

Method C: To a solution of epoxide (0.5–0.75 mmol) in anhydrous toluene (10 mL) under an Ar atmosphere, 50% Deoxofluor in toluene (4 equiv.) and the given amount of TiCl_4_ was added, and the solution was stirred at 20 °C for the time given in the schemes. The solution was then diluted with CH_2_Cl_2_ (30 mL), the organic layer was washed with a saturated aqueous NaHCO_3_ solution (2 × 20 mL), dried (Na_2_SO_4_), and concentrated. The crude product was purified by column chromatography on silica gel (*n*-hexane/EtOAc).

Method D: To a solution of epoxide (0.5 mmol) in anhydrous 1,4-dioxane (10 mL) under an Ar atmosphere, one drop of anhydrous EtOH (or without the addition of EtOH, see the schemes) was added, followed by XtalFluor-E (1 equiv.). The reaction mixture was kept at a reflux temperature for the time given in the schemes. The solution after cooling was diluted with CH_2_Cl_2_ (30 mL), and the organic layer was washed with a saturated aqueous NaHCO_3_ solution (2 × 20 mL), dried (Na_2_SO_4_), and concentrated. The crude product was purified by column chromatography on silica gel (*n*-hexane/EtOAc).

Ethyl (1R*,2R*,3R*,5S*) 2-benzamido-6-oxabicyclo[3.1.0]hexane-3-carboxylate **(±)-2**. White solid; yield 97%; *R*_f_ = 0.48 (*n*-hexane/EtOAc 1:2); m.p. 85–88 °C. ^1^H-NMR (400 MHz, CDCl_3_, TMS) δ = 1.27 (t, *J* = 7.12 Hz, 3H, CH_3_), 2.01–2.10 (m, 1H, CH_2_), 2.75–2.83 (m, 2H, CH_2_ and H-3), 3.49–3.53 (m, 1H, H-1), 3.67–3.71 (m, 1H, H-5), 4.10–4.26 (m, 2H, OCH_2_), 4.89–4.96 (m, 1H, H-2), 7.42–7.55 (m, 3H, Ar-H), 7.85–7.91 (m, 2H, Ar-H), 8.63–8.79 (brs, 1H, N-H). ^13^C-NMR (100 MHz, CDCl_3_, TMS) δ = 14.4, 30.8, 37.5, 53.3, 54.0, 57.5, 61.6, 127.5, 129.0, 132.0, 134.4, 167.6, 174.8. MS (ESI, pos) *m/z* 298 (M + Na). Anal. Calcd. for C_15_H_17_NO_4_: C, 65.44; H, 6.22; N, 5.09. Found: C, 65.11; H, 5.88; N, 4.81.

Ethyl (1R*,2R*,3S*,5S*) 2-benzamido-6-oxabicyclo[3.1.0]hexane-3-carboxylate **(±)-6**. White solid; yield 97%; *R*_f_ = 0.62 (*n*-hexane/EtOAc 1:2); m.p. 108–111 °C. ^1^H-NMR (400 MHz, CDCl_3_, TMS) δ = 1.20 (t, *J* = 7.14 Hz, 3H, CH_3_), 2.10–2.21 (m, 2H, CH_2_), 2.40–2.52 (m, 1H, H-3), 3.56–3.62 (m, 1H, H-1), 3.69–3.76 (m, 1H, H-5), 4.07–4.22 (m, 2H, OCH_2_), 4.83–4.92 (m, 1H, H-2), 6.34 (d, *J* = 7.64 Hz, 1H, N-H), 7.41–7.48 (m, 2H, Ar-H), 7.49–7.55 (m, 1H, Ar-H), 7.76–7.82 (m, 2H, Ar-H). ^13^C-NMR (100 MHz, CDCl_3_, TMS) δ = 14.5, 30.7, 44.0, 55.3, 55.5, 58.1, 61.6, 127.4, 129.0, 132.1, 134.5, 167.9, 173.3. MS (ESI, pos) *m/z* 276 (M + 1). Anal. Calcd. for C_15_H_17_NO_4_: C, 65.44; H, 6.22; N, 5.09. Found: C, 65.10; H, 6.86; N, 4.80.

Ethyl (1S*,3R*,4S*,6R*) 4-benzamido-7-oxabicyclo[4.1.0]heptane-3-carboxylate **(±)-10**. White solid; yield 69%; *R*_f_ = 0.47 (*n*-hexane/EtOAc 1:2); m.p. 100–109 °C. ^1^H-NMR (400 MHz, CDCl_3_, TMS) δ = 1.31 (t, *J* = 7.12 Hz, 3H, CH_3_), 2.16–2.26 (m, 1H, CH_2_), 2.27–2.34 (m, 2H, CH_2_), 2.57–2.65 (m, 1H, CH_2_), 2.70–2.79 (m, 1H, H-3), 3.26–3.32 (m, 2H, H-1 and H-6), 4.12–4.27 (m, 2H, OCH_2_), 4.60–4.69 (m, 1H, H-4), 7.34 (d, *J* = 9.20 Hz, 1H, N–H), 7.41–7.48 (m, 2H, Ar-H), 7.48–7.55 (m, 1H, Ar-H), 7.75–7.82 (m, 2H, Ar-H). ^13^C-NMR (100 MHz, CDCl_3_, TMS) δ = 14.5, 25.1, 29.1, 40.6, 44.5, 51.5, 52.1, 61.4, 127.4, 128.9, 131.8, 134.9, 166.8, 173.4. MS (ESI, pos) *m/z* 290 (M + 1). Anal. Calcd. for C_16_H_19_NO_4_: C, 66.42; H, 6.62; N, 4.84. Found: C, 66.09; H, 6.29; N, 4.50.

Ethyl (1S*,3S*,4S*,6R*) 4-benzamido-7-oxabicyclo[4.1.0]heptane-3-carboxylate **(±)-13**. White solid; yield 91%; *R*_f_ = 0.48 (*n*-hexane/EtOAc 1:2); m.p. 108–119 °C. ^1^H-NMR (400 MHz, CDCl_3_, TMS) δ = 1.30 (t, *J* = 7.14 Hz, 3H, CH_3_), 2.06–2.14 (m, 1H, CH_2_), 2.22–2.30 (m, 1H, CH_2_), 2.32–2.40 (m, 1H, CH_2_), 2.45–2.54 (m, 1H, CH_2_), 2.86–2.96 (m, 1H, H-3), 3.31–3.36 (m, 1H, H-6), 3.38–3.43 (m, 1H, H-1), 4.22 (q, *J* = 7.06 Hz, 2H, OCH_2_), 4.61–4.70 (m, 1H, H-4), 7.04 (d, *J* = 9.44 Hz, 1H, N-H), 7.44–7.50 (m, 2H, Ar-H), 7.51–7.57 (m, 1H, Ar-H), 7.77–7.84 (m, 2H, Ar-H). ^13^C-NMR (100 MHz, CDCl_3_, TMS) δ = 14.6, 23.7, 28.2, 40.9, 45.3, 51.8, 52.6, 61.5, 127.3, 129.0, 132.0, 134.6, 166.7, 173.4. MS (ESI, pos) *m/z* 290 (M + 1). Anal. Calcd. for C_16_H_19_NO_4_: C, 66.42; H, 6.62; N, 4.84. Found: C, 66.11; H, 6.90; N, 4.49.

*Ethyl 2-benzamido-4-fluorocyclopent-1-enecarboxylate* **(±)-3**. Yellowish solid; yield 37%; *R*_f_ = 0.52 (*n*-hexane/EtOAc 5:1); m.p. 72–82 °C. ^1^H-NMR (400 MHz, CDCl_3_, TMS) δ = 1.34 (t, *J* = 7.12 Hz, 3H, CH_3_), 2.82–2.85 (m, 1H, H-5), 2.90–2.94 (m, 1H, H-5), 3.46–3.81 (m, 2H, H-3), 4.22–4.32 (m, 2H, OCH_2_), 5.20–5.39 (m, 1 H, H-4), 7.47–7.53 (m, 2H, Ar-H), 7.54–7.60 (m, 1H, Ar-H), 7.92–7.98 (m, 2H, Ar-H), 11.26–11.36 (s, 1H, N-H). ^19^F-NMR (376 MHz, CDCl_3_) δ = −168.91. ^13^C-NMR (100 MHz, CDCl_3_, TMS) δ = 14.8, 30.1, 37.05 and 37.29 (^2^*J* = 24.00 Hz), 42.69 and 42.94 (^2^*J* = 25.25 Hz), 60.7, 90.28 and 92.03 (^1^*J* = 176.35 Hz), 105.7, 128.0, 129.3, 132.9, 133.7, 151.9, 165.2. MS (ESI, pos) *m/z* 577 (2M + Na). Anal. Calcd. for C_15_H_16_FNO_3_: C, 64.97; H, 5.82; N, 5.05. Found: C, 64.66; H, 5.48; N, 4.77.

Ethyl (1R*,2R*,3R*,4R*) 2-benzamido-4-fluoro-3-hydroxycyclopentane-carboxylate **(±)-4**. Yellow solid; yield 26%; *R*_f_ = 0.62 (*n*-hexane/EtOAc 1:1); m.p. 96–104 °C. ^1^H-NMR (400 MHz, CDCl_3_, TMS) δ = 1.13 (t, *J* = 7.14 Hz, 3H, CH_3_), 2.19–2.36 (m, 1H, CH_2_), 2.46–2.65 (m, 1H, CH_2_), 3.58 (dt, *J* = 5.41 Hz and 8.91 Hz, 1H, H-1), 4.09 (q, *J* = 7.13 Hz, 2H, OCH_2_), 4.26 (dd, *J* = 4.78 Hz and 10.22 Hz, 1H, H-3), 4.84–4.92 (m, 1H, H-2), 4.95–5.12 (m, 1H, H-4), 7.05 (d, *J* = 7.36 Hz, 1H, N-H), 7.39–7.46 (m, 2H, Ar-H), 7.47–7.54 (m, 1H, Ar-H), 7.73–7.79 (m, 2H, Ar-H). ^19^F-NMR (376 MHz, CDCl_3_) δ = −175.66. ^13^C-NMR (100 MHz, CDCl_3_, TMS) δ = 14.4, 34.25 and 34.49 (^2^*J* = 23.65 Hz), 44.78, 53.51, 62.36, 76.63 and 76.91 (^2^*J* = 27.70 Hz), 96.10 and 97.88 (^1^*J* = 179.42 Hz), 127.4, 129.1, 132.3, 177.4. MS (ESI, pos) *m/z* 296 (M + 1). Anal. Calcd. for C_15_H_18_FNO_4_: C, 61.01; H, 6.14; N, 4.74. Found: C, 60.70; H, 5.79; N, 4.38.

Ethyl (3aR*,4R*,6S*,6aR*)-6-hydroxy-2-phenyl-4,5,6,6a-tetrahydro-3aH-cyclo-penta[d]oxazole-4-carboxylate **(±)-5**. White solid; yield 57%; *R*_f_ = 0.35 (*n*-hexane/EtOAc 1:2); m.p. 130–134 °C. ^1^H-NMR (400 MHz, *d*_6_-DMSO, TMS) δ = 1.23 (t, *J* = 7.08 Hz, 3H, CH_3_), 1.51–1.63 (m, 1H, CH_2_), 1.73–1.87 (m, 1H, CH_2_), 2.97–3.06 (m, 1H, H-4), 4.00–4.16 (m, 3H, OCH_2_ and H-3a), 4.74–4.84 (m, 2H, H-6 and H-6a), 5.05 (d, *J* = 7.12 Hz, 1H, O-H), 7.45–7.51 (m, 2H, Ar-H), 7.53–7.59 (m, 1H, Ar-H), 7.83–7.88 (m, 2H, Ar-H). ^13^C-NMR (100 MHz, CDCl_3_, TMS) δ = 14.7, 31.9, 46.3, 61.2, 71.8, 74.2, 83.2, 127.1, 128.8, 129.0, 132.2, 165.0, 171.0. MS (ESI, pos) *m/z* 276 (M + 1). Anal. Calcd. for C_15_H_17_NO_4_: C, 65.44; H, 6.22; N, 5.09. Found: C, 65.08; H, 5.87; N, 4.73.

Ethyl (1S*,2R*,3S*,4R*) 2-benzamido-3,4-difluorocyclopentanecarboxylate **(±)-7**. White solid; yield 15%; *R*_f_ = 0.39 (*n*-hexane/EtOAc 2:1); m.p. 152–161 °C. ^1^H-NMR (400 MHz, CDCl_3_, TMS) δ = 1.26 (t, *J* = 7.12 Hz, 3H, CH_3_), 2.31–2.46 (m, 1H, CH_2_), 2.48–2.65 (m, 1H, CH_2_), 3.40–3.49 (m, 1H, H-1), 4.19 (q, *J* = 7.06 Hz, 2H, OCH_2_), 4.33–4.47 (m, 1H, H-2), 5.07–5.26 (m, 1H, H-4), 5.40–5.63 (m, 1H, H-3), 6.70 (d, *J* = 5.92 Hz, 1H, N-H), 7.42–7.48 (m, 2H, Ar-H), 7.50–7.56 (m, 1H, Ar-H), 7.73–7.78 (m, 2H, Ar-H). ^19^F-NMR (376 MHz, CDCl_3_) δ = −196.34, −203.13. ^13^C-NMR (100 MHz, CDCl_3_, TMS) δ = 14.6, 31.01 and 31.22 (^2^*J* = 21.04 Hz), 41.83 and 41.89 (^3^*J* = 6.13 Hz), 57.96 and 58.20 (^2^J = 24.37 Hz), 61.81, 89.52 and 89.68 and 91.36 and 91.52 (^1^*J* = 185.10 Hz, ^2^*J* = 15.14 Hz), 91.91 and 92.06 and 93.84 and 94.00 (^1^*J* = 194.95 Hz, ^2^*J* = 15.62 Hz), 127.3, 129.1, 132.4, 134.2, 168.5, 173.6. MS (ESI, pos) *m/z* 298 (M + 1). Anal. Calcd. for C_15_H_17_F_2_NO_3_: C, 60.60; H, 5.76; N, 4.71. Found: C, 60.25; H, 5.39; N, 4.40.

Ethyl (3aR*,4S*,6S*,6aR*)-6-hydroxy-2-phenyl-4,5,6,6a-tetrahydro-3aH-cyclo-penta[d]oxazole-4-carboxylate **(±)-8**. White solid; yield 85%; *R*_f_ = 0.57 (*n*-hexane/EtOAc 1:2); m.p. 54–64 °C. ^1^H-NMR (400 MHz, CDCl_3_, TMS) δ = 1.29 (t, *J* = 7.14 Hz, 3H, CH_3_), 1.74–1.84 (m, 1H, CH_2_), 2.18–2.26 (m, 1H, CH_2_), 3.09–3.16 (m, 1H, H-4), 4.19 (q, *J* = 7.14 Hz, 2H, OCH_2_), 4.42–4.50 (m, 1H, H-6), 4.93–5.00 (m, 1H, H-3a), 5.01–5.08 (m, 1H, H-6a), 7.41–7.48 (m, 2H, Ar-H), 7.49–7.58 (m, 1H, Ar-H), 7.99–8.06 (m, 2H, Ar-H). ^13^C-NMR (100 MHz, CDCl_3_, TMS) δ = 14.6, 34.0, 48.5, 61.5, 61.6, 73.1, 73.8, 83.6, 127.0, 127.5, 128.9, 132.4, 164.7, 174.1. MS (ESI, pos) *m/z* 276 (M + 1). Anal. Calcd. for C_15_H_17_NO_4_: C, 65.44; H, 6.22; N, 5.09. Found: C, 65.07; H, 5.85; N, 4.74.

Ethyl (1R*,2S*,4S*,5R*) 2-benzamido-4,5-difluorocyclohexanecarboxylate **(±)-11**. White solid; yield 76%; *R*_f_ = 0.44 (*n*-hexane/EtOAc 2:1); m.p. 158–162 °C. ^1^H-NMR (400 MHz, CDCl_3_, TMS) δ = 1.31 (t, *J* = 7.14 Hz, 3H, CH_3_), 2.17–2.48 (m, 4H, CH_2_), 3.16–3.24 (m, 1H, H-1), 4.17–4.30 (m, 2H, OCH_2_), 4.61–4.86 (m, 2H, H-5 and H-2), 4.88–5.09 (m, 1H, H-4), 6.99 (d, *J* = 6.68 Hz, 1H, N-H), 7.42–7.49 (m, 2H, Ar-H), 7.50–7.57 (m, 1H, Ar-H), 7.74–7.80 (m, 2H, Ar-H). ^19^F-NMR (376 MHz, CDCl_3_) δ = −192.2, −201.8. ^13^C-NMR (100 MHz, CDCl_3_, TMS) δ = 14.5, 27.48 and 27.70 (^2^*J* = 22.10 Hz), 31.99 and 32.19 (^2^*J* = 20.16 Hz), 42.0, 44.2, 61.8, 86.83 and 87.01 and 88.64 and 88.82 (^1^*J* = 182.07 Hz, ^2^*J* = 18.31 Hz), 87.2 and 87.42 and 89.03 and 89.21 (^1^*J* = 179.59 Hz, ^2^*J* = 17.45 Hz), 127.3, 129.0, 132.1, 134.6, 167.1, 173.7. MS: (ESI, pos) *m/z* = 312 (M +1). Anal. Calcd. for C_16_H_19_F_2_NO_3_: C, 61.73; H, 6.15; N, 4.50. Found: C, 61.40; H, 5.79; N, 4.16.

Ethyl (1R*,5S*,6R*,8S*) 8-hydroxy-3-phenyl-2-oxa-4-azabicyclo[3.3.1]non-3-ene-6-carboxylate **(±)-12**. Yellow oil; yield 37%; *R*_f_ = 0.49 (*n*-hexane/EtOAc 1:3). ^1^H-NMR (400 MHz, CDCl_3_, TMS) δ = 1.34 (t, *J* = 7.12 Hz, 3H, CH_3_), 1.58–1.71 (m, 1H, CH_2_), 1.78–1.85 (m, 1H, CH_2_), 2.15–2.27 (m, 2H, CH_2_), 2.75–2.83 (m, 1H, H-6), 3.80–3.87 (m, 1H, H-8), 4.27 (q, *J* = 7.10 Hz, 2H, OCH_2_), 4.34–4.38 (m, 1H, H-5), 4.70–4.74 (m, 1H, H-1), 7.37–7.43 (m, 2H, Ar-H), 7.43–7.50 (m, 1H, Ar-H), 7.93–7.97 (m, 2H, Ar-H). ^13^C-NMR (100 MHz, CDCl_3_, TMS) δ = 14.6, 27.1, 28.3, 30.1, 47.2, 48.2, 61.3, 73.3, 74.8, 127.7, 128.4, 131.1, 131.8, 133.5, 157.9, 172.3. MS (ESI, pos) *m/z* 290 (M + 1). Anal. Calcd. for C_16_H_19_NO_4_: C, 66.42; H, 6.62; N, 4.84. Found: C, 66.09; H, 6.92; N, 4.50.

Ethyl (1S*,2S*,4S*,5R*) 2-benzamido-4,5-difluorocyclohexanecarboxylate **(±)-15**. Yellowish brown solid; yield 10%; *R*_f_ = 0.53 (*n*-hexane/EtOAc 2:1); m.p. 42–51 °C. ^1^H-NMR (400 MHz, CDCl_3_, TMS) δ = 1.16 (t, *J* = 7.12 Hz, 3H, CH_3_), 1.83–2.14 (m, 1H, CH_2_) 2.15–2.38 (m, 1H, CH_2_) or 1.83–2.38 (m, 1H, CH_2_), 2.42–2.58 (m, 1H, CH_2_), 2.80–2.95 (m, 1H, H-1), 4.05–4.20 (m, 2H, OCH_2_), 4.43–4.57 (m, 1H, H-2), 4.75–4.97 (m, 2H, H-4 and H-5), 6.31 (d, *J* = 7.56 Hz, 1H, N-H), 7.36–7.45 (m, 2H, Ar-H), 7.46–7.53 (m, 1H, Ar-H), 7.69–7.78 (m, 2H, Ar-H). ^19^F-NMR (376 MHz, CDCl_3_) δ = −192.71. ^13^C-NMR (100 MHz, CDCl_3_, TMS) δ = 14.5, 28.66 and 28.86 (^2^*J* = 19.67 Hz), 32.67 and 32.87 (^2^*J* = 20.27 Hz), 43.4, 46.0, 61.7, 84.78 and 85.08 and 86.47 and 86.78 (^1^*J* = 170.33 Hz, ^2^*J* = 30.58 Hz), 85.95 and 86.29 and 87.69 and 88.03 (^1^*J* = 175.08 Hz, ^2^*J* = 34.96 Hz), 127.3, 129.0, 132.0, 134.7, 167.3, 172.9. MS (ESI, pos) *m/z* 312 (M + 1). Anal. Calcd. for C_16_H_19_F_2_NO_3_: C, 61.73; H, 6.15; N, 4.50. Found: C, 61.39; H, 5.81; N, 4.87.

Ethyl (1R*,5S*,6S*,8S*) 8-hydroxy-3-phenyl-2-oxa-4-azabicyclo[3.3.1]non-3-ene-6-carboxylate **(±)-14**. Yellow oil; yield 46%; *R*_f_ = 0.47 (*n*-hexane/EtOAc 2:3); ^1^H-NMR (400 MHz, CDCl_3_, TMS) δ = 1.29 (t, *J* = 7.14 Hz, 3H, CH_3_), 1.39–1.50 (m, 1H, CH_2_), 1.84–1.91 (m, 1H, CH_2_), 1.98–2.06 (m, 1H, CH_2_), 2.30–2.38 (m, 1H, CH_2_), 3.09–3.15 (m, 1H, H-6), 4.05–4.12 (m, 1H, H-8), 4.15–4.24 (m, 3H, OCH_2_ and H-5), 4.67–4.73 (m, 1H, H-1), 7.35–7.42 (m, 2H, Ar-H), 7.42–7.47 (m, 1H, Ar-H), 7.92–7.98 (m, 2H, Ar-H). ^13^C-NMR (100 MHz, CDCl_3_, TMS) δ =14.6, 24.5, 27.9, 45.8, 47.6, 61.4, 70.6, 75.4, 127.6, 128.5, 131.3, 133.5, 158.4, 173.3. MS (ESI, pos) *m/z* 290 (M + 1). Anal. Calcd. for C_16_H_19_NO_4_: C, 66.42; H, 6.62; N, 4.84. Found: C, 66.07; H, 6.29; N, 4.51.

Ethyl (1S*,2S*,4S*,5S*) 2-benzamido-4-fluoro-5-hydroxycyclohexanecarboxylate **(±)-16**. Brown oil; yield 13%; *R*_f_ = 0.53 (*n*-hexane/EtOAc 1:2). ^1^H-NMR (400 MHz, CDCl_3_, TMS) δ = 1.16 (t, *J* = 7.10 Hz, 3H, CH_3_), 1.91–2.40 (m, 4H, CH_2_), 2.97–3.07 (m, 1H, H-1), 4.03–4.16 (m, 3H, OCH_2_ and H-5), 4.45–4.56 (m, 1H, H-2), 4.62–4.81 (m, 1H, H-4), 6.47 (d, *J* = 7.96 Hz, 1H, N-H), 7.34–7.43 (m, 2H, Ar-H), 7.43–7.51 (m, 1H, Ar-H), 7.68–7.76 (m, 2H, Ar-H). ^19^F-NMR (376 MHz, CDCl_3_) δ = −187.14. ^13^C-NMR (100 MHz, CDCl_3_, TMS) δ = 14.5, 30.5, 32.11 and 32.31 (^2^*J* = 20.31 Hz), 43.2, 46.6, 61.5, 65.94 and 66.21 (^2^*J* = 27.35 Hz), 89.60 and 91.32 (^1^*J* = 172.66 Hz), 127.3, 128.9, 131.9, 134.8, 167.5, 173.9. MS (ESI, pos) *m/z* 310 (M + 1). Anal. Calcd. for C_16_H_20_FNO_4_: C, 62.12; H, 6.52; N, 4.53. Found: C, 61.81; H, 6.21; N, 4.17.

## 4. Conclusions

A novel synthetic procedure has been developed for the access of some highly functionalized fluorine-containing alicyclic scaffolds. The synthetic procedure was based on the ring opening reaction of functionalized cycloalkane-fused oxiranes with both XtalFluor-E and Deoxofluor as fluoride sources. Through an investigation with various experimental conditions, the opening of the three-membered heterocyclic ring with fluoride was found to be highly substrate-dependent. However, this substrate directable synthetic investigation of an epoxide opening with fluoride using XtalFluor-E or Deoxofluor needs further study in order to achieve higher yields. Synthetic investigations using other highly functionalized model compounds under various experimental conditions are currently being studied in our laboratory.

## Supplementary Materials

Supplementary materials can be accessed at: http://www.mdpi.com/1420-3049/21/11/1493/s1.

## References

[1] Chawla R., Singh A.K., Yadav L.D.S. (2013). Organocatalysis in synthesis and reactions of epoxides and aziridines. RSC Adv..

[2] Meninno S., Lattanzi A. (2016). Organocatalytic asymmetric reactions of epoxides: Recent progress. Chem. Eur. J..

[3] Krake S.H., Bergmeier S.C. (2010). Inter- and intramolecular reactions of epoxides and aziridines with π-nucleophiles. Tetrahedron.

[4] Zhu Y., Wang Q., Cornwall R.G., Shi Y. (2014). Organocatalytic asymmetric epoxidation and aziridination of olefins and their synthetic applications. Chem. Rev..

[5] Kiss L., Remete A.M., Nonn M., Fustero S., Sillanpaa R., Fülöp F. (2016). Substrate-dependent fluorinations of highly functionalized cycloalkanes. Tetrahedron.

[6] Kiss L., Forró E., Fülöp F. (2012). Selective syntheses of novel highly functionalized beta-aminocyclohexanecarboxylic acids. Tetrahedron.

[7] Wang P.A. (2013). Organocatalyzed enantioselective desymmetrization of aziridines and epoxides. Beilstein J. Org. Chem..

[8] Wang J., Sánchez-Roselló M., Aceña J.L., del Pozo C., Sorochinsky A.E., Fustero S., Soloshonok V.A., Liu H. (2014). Fluorine in pharmaceutical industry: Fluorine-containing drugs introduced to the market in the last decade (2001–2011). Chem. Rev..

[9] O’Hagan D. (2010). Fluorine in health care: Organofluorine containing blockbuster drugs. J. Fluor. Chem..

[10] Mikami K., Fustero S., Sanchez-Rosello M., Acena J.L., Soloshonok V.A., Sorochinsky A. (2011). Synthesis of fluorinated beta-amino acids. Synthesis.

[11] Kiss L., Forró E., Fustero S., Fülöp F. (2011). Selective synthesis of new fluorinated alicyclic β-amino ester stereoisomers. Eur. J.Org. Chem..

[12] Nonn M., Kiss L., Haukka M., Fustero S., Fülöp F. (2015). A Novel and selective fluoride opening of aziridines by XtalFluor-E. Synthesis of Fluorinated Diamino Acid Derivatives. Org. Lett..

[13] Kiss L., Nonn M., Forró E., Sillanpää R., Fustero S., Fülöp F. (2014). A selective synthesis of fluorinated cispentacin derivatives. Eur. J. Org. Chem..

[14] Wu J. (2014). Review of recent advances in nucleophilic C–F bond-forming reactions at SP3 centers. Tetrahedron Lett..

[15] Ma J.A., Cahard D. (2008). Asymmetric fluorination, trifluoromethylation, and perfluoroalkylation reactions. Chem. Rev..

[16] Champagne P.A., Desroches J., Hamel J.D., Vandamme M., Paquin J.F. (2015). Monofluorination of organic compounds: 10 years of innovation. Chem. Rev..

[17] Liang T., Neumann C.N., Ritter T. (2013). Introduction of fluorine and fluorine-containing functional groups. Angew. Chem. Int. Ed..

[18] Husstedt W.S., Wiehle S., Stillig C., Bergander C., Grimme S., Haufe G. (2011). Synthesis and preferred conformations of all regio- and diastereoisomeric methyl 2,3-fluorohydroxyalkanoates. Eur. J. Org. Chem..

[19] O’Hagan D. (2012). Organofluorine chemistry: Synthesis and conformation of vicinal fluoromethylene motifs. J. Org. Chem..

[20] Hunter L., Jolliffe K.A., Jordan M.J.T., Jensen P., Macquart R.B. (2011). Synthesis and conformational analysis of alfa,beta-difluoro-gamma-amino acid derivatives. Chem. Eur. J..

[21] Bykova T., Al-Maharik N., Slawin A.M.Z., O’Hagan D. (2016). Multicomponent reactions of methyl substituted *all-cis* tetrafluorocyclohexane aldehydes. Org. Biomol. Chem..

[22] Fraile J.M., Mayoral J.A., Salvatella L. (2014). Theoretical study on the BF_3_-catalyzed meinwald rearrangement reaction. J. Org. Chem..

[23] Cresswell A.J., Davies S.G., Lee J.A., Roberts P.M., Russell A.J., Thomson J.E., Tyte M. (2010). β-Fluoroamphetamines via the stereoselective synthesis of benzylic fluorides. J. Org. Lett..

[24] Cresswell A.J., Davies S.G., Lee J.A., Morris M.J., Roberts P.M., Thomson J.E. (2011). Ring-opening hydrofluorination of 2,3- and 3,4-epoxy amines by HBF_4_3OEt_2_: Application to the asymmetric synthesis of (*S*,*S*)-3-deoxy-3-fluorosafingol. J. Org. Chem..

[25] Cresswell A.J., Davies S.G., Lee J.A., Morris M.J., Roberts P.M., Thomson J.E. (2012). Diastereodivergent hydroxyfluorination of cyclic and acyclic allylic amines: Synthesis of 4-deoxy-4-fluorophytosphingosines. J. Org. Chem..

[26] Kondapi V.P.K., Soueidan O.M., Hosseini S.N., Jabari N., West F.G. (2016). Efficient and easy access to optically pure tetrasubstituted tetrahydrofurans via stereoselective opening of C2-symmetric epoxide and aziridine rings. Eur. J. Org. Chem..

[27] Yan N., Fang Z., Liu Q.Q., Guo X.H., Hu X.G. (2016). Conformation-induced regioselective and divergent opening of epoxides by fluoride: Facile access to hydroxylated fluoro-piperidines. Org. Biomol. Chem..

[28] Bruns S., Haufe G. (2000). Enantioselective introduction of fluoride into organic compounds. First asymmetric ring opening of epoxides by hydrofluorinating reagents. J. Fluor. Chem..

[29] Haufe G., Bruns S., Runge M. (2001). Enantioselective ring opening of epoxides by HF-reagents. Asymmetric synthesis of fluorolactones. J. Fluor. Chem..

[30] Althaus M., Togni A., Mezzetti A. (2009). Asymmetric oxidative alfa-fluorination of 2-alkylphenylacetaldehydes with AgHF_2_ and ruthenium/PNNP catalysts. J. Fluor. Chem..

[31] Kalow J.A., Doyle A.G. (2010). Enantioselective ring opening of epoxides by fluoride anion promoted by a cooperative dual-catalyst system. J. Am. Chem. Soc..

[32] Kalow J.A., Doyle A.G. (2011). Mechanistic investigations of cooperative catalysis in the enantioselective fluorination of epoxides. J. Am. Chem. Soc..

[33] Kiss L., Fülöp F. (2014). Synthesis of carbocyclic and heterocyclic β-aminocarboxylic acids. Chem. Rev..

[34] Kiss L., Forró E., Sillanpää R., Fülöp F. (2007). Diastereo- and enantioselective synthesis of orthogonally protected 2,4-diaminocyclopentanecarboxylates: A flip from β-amino- to β,γ-diaminocarboxylates. J. Org. Chem..

[35] Nonn M., Kiss L., Forró E., Mucsi Z., Fülöp F. (2011). Synthesis of novel isoxazoline-fused cyclic beta-amino esters by regio- and stereoselective 1,3-dipolar cycloaddition. Tetrahedron.

[36] Cherepanova M., Kiss L., Forró E., Fülöp F. (2014). A De Novo Stereocontrolled Approach to syn- and anti-Disubstituted Acyclic β-2,3-Amino Acid Enantiomers. Eur. J. Org. Chem..

[37] L’Heureux A., Beaulieu F., Bennett C., Bill D.R., Clayton S., LaFlamme F., Mirmehrabi M., Tadayon S., Tovell D., Couturier M. (2010). Aminodifluorosulfinium Salts: Selective Fluorination Reagents with Enhanced Thermal Stability and Ease of Handling. J. Org. Chem..

[38] Lal G.S., Pez G.P., Pesaresi R.J., Prozonic F.M., Cheng H. (1999). Bis(2-methoxyethyl)aminosulfur Trifluoride: A New Broad-Spectrum Deoxofluorinating Agent with Enhanced Thermal Stability. J. Org. Chem..

[39] Kiss L., Forró E., Martinek T.A., Bernáth G., de Kimpe N., Fülöp F. (2008). Stereoselective synthesis of hydroxylated beta-aminocyclohexanecarboxylic acids. Tetrahedron.

[40] Cherepanova M., Kiss L., Fülöp F. (2014). Stereocontrolled transformation of cyclohexene beta-amino esters into syn- or anti-difunctionalized acyclic beta2,3-amino acid derivatives. Tetrahedron.

