# Supplementary material for: A Stereocontrolled Protocol to Highly Functionalized Fluorinated Scaffolds through a Fluoride Opening of Oxiranes
Source: Molecules. 2016 Nov 17;21(11):1493. doi: 10.3390/molecules21111493 (PMC6274065; doi:10.3390/molecules21111493)
Supplement: Supplementary file 1 [file molecules-21-01493-s001.pdf]

Supplementary Materials: A Stereocontrolled Protocol to Highly Functionalized Fluorinated Scaffolds through a Fluoride Opening of Oxiranes

Attila Márió Remete, Melinda Nonn, Santos Fustero, Ferenc Fülöp and Loránd Kiss

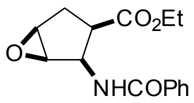

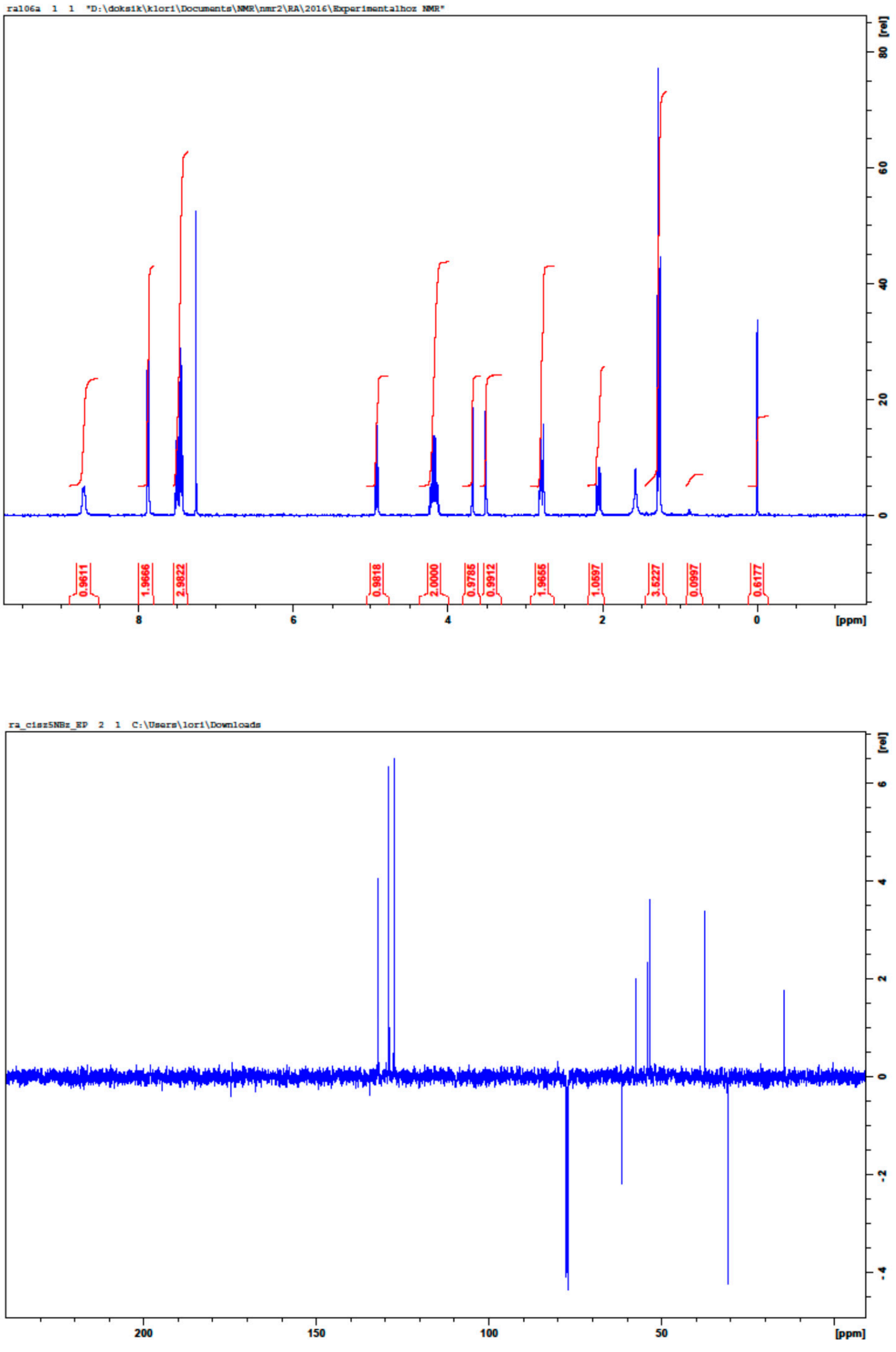

Figure 1. ${}^{1}H$-NMR and ${}^{13}C$-NMR spectra of compound **(±)-2**.

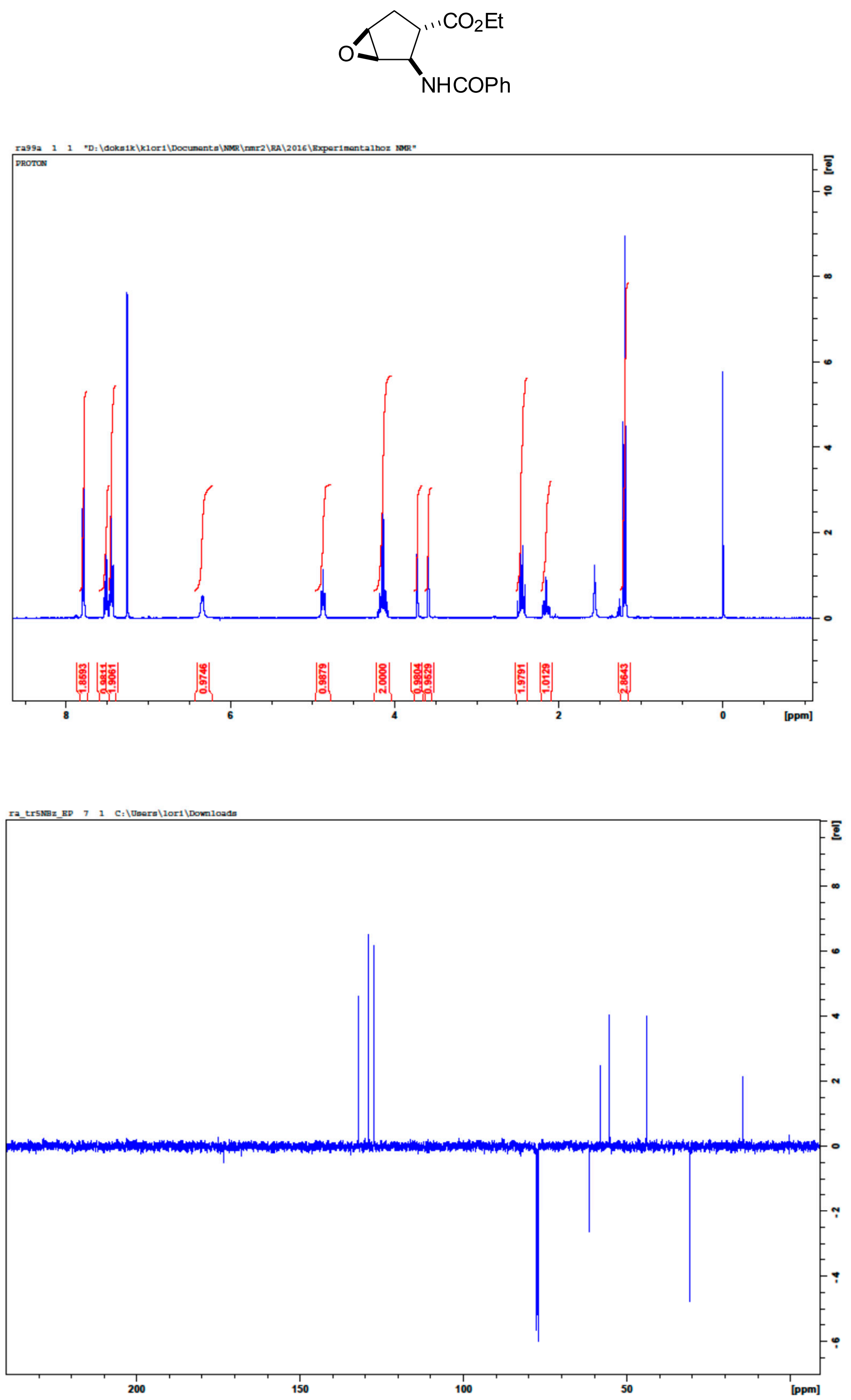

Figure 2. ^{1}H-NMR and ^{13}C-NMR spectra of compound **(±)-6.**

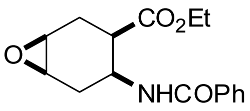

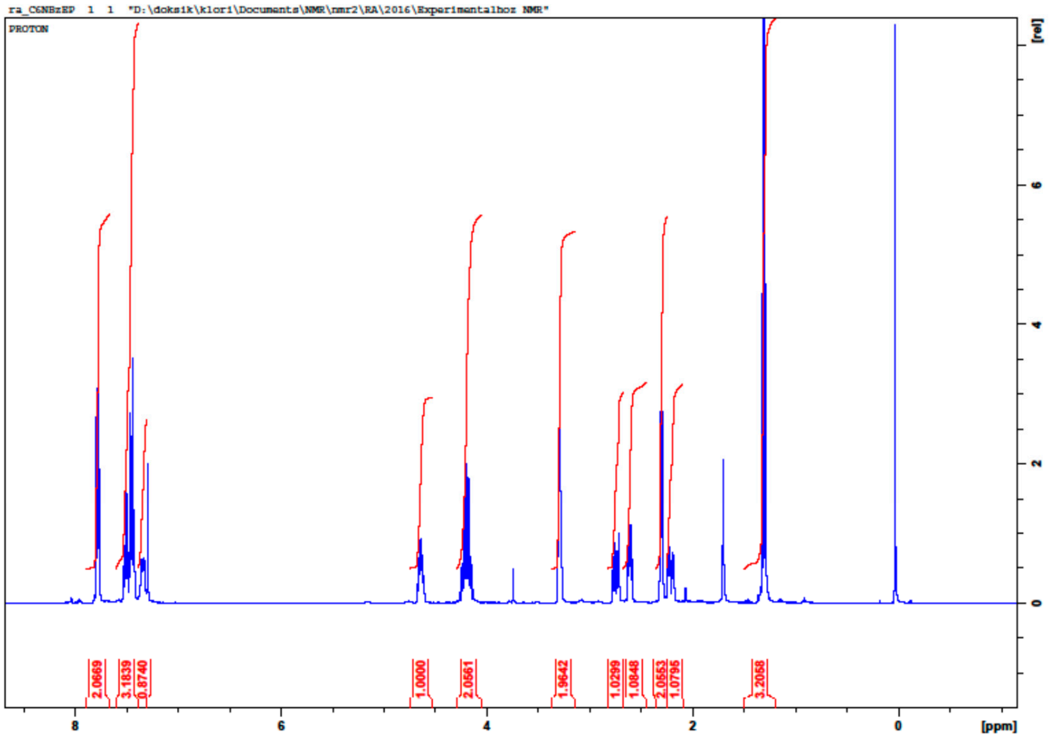

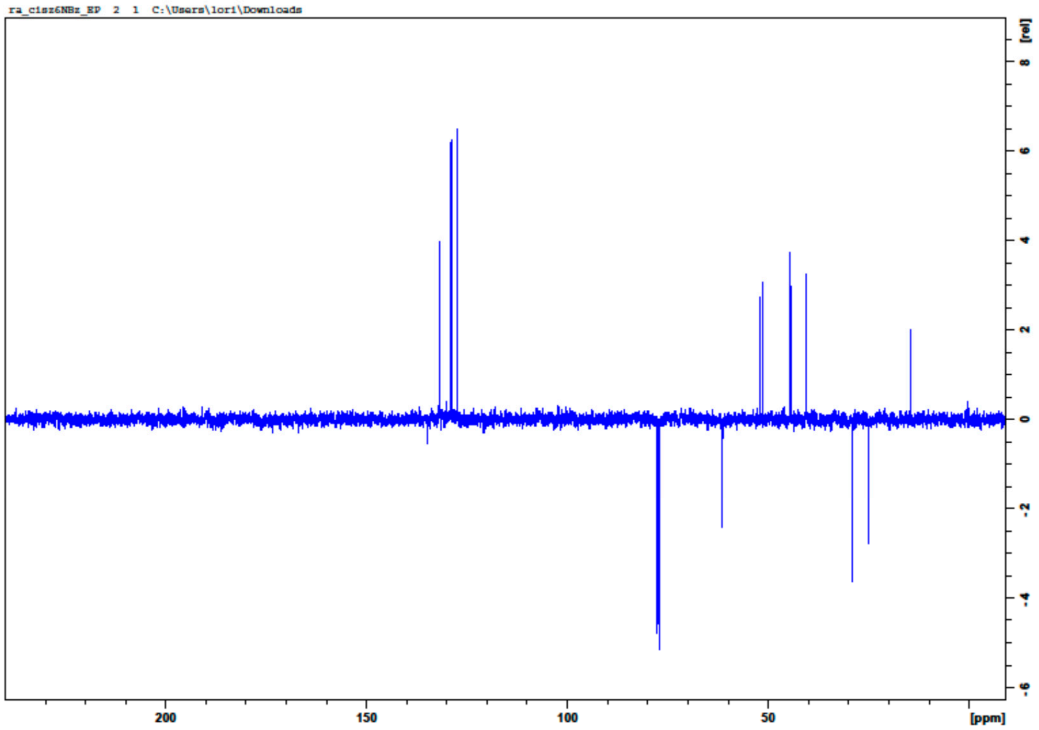

Figure 3. ^{1}H-NMR and ^{13}C-NMR spectra of compound **(±)-10**.

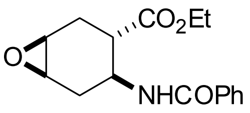

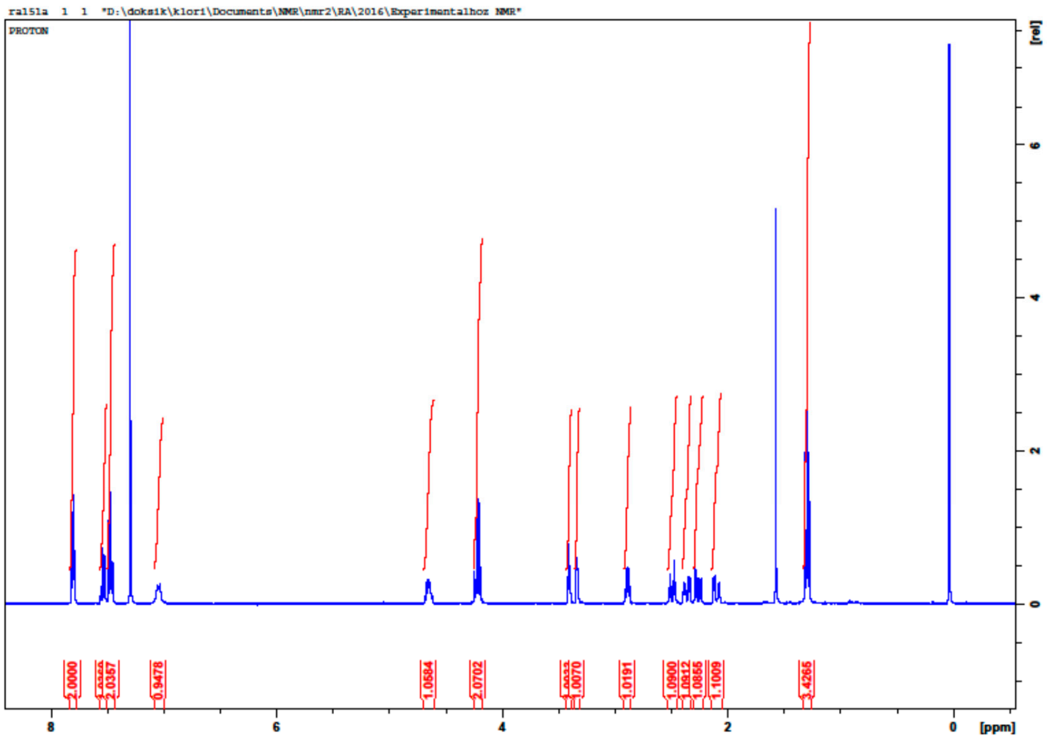

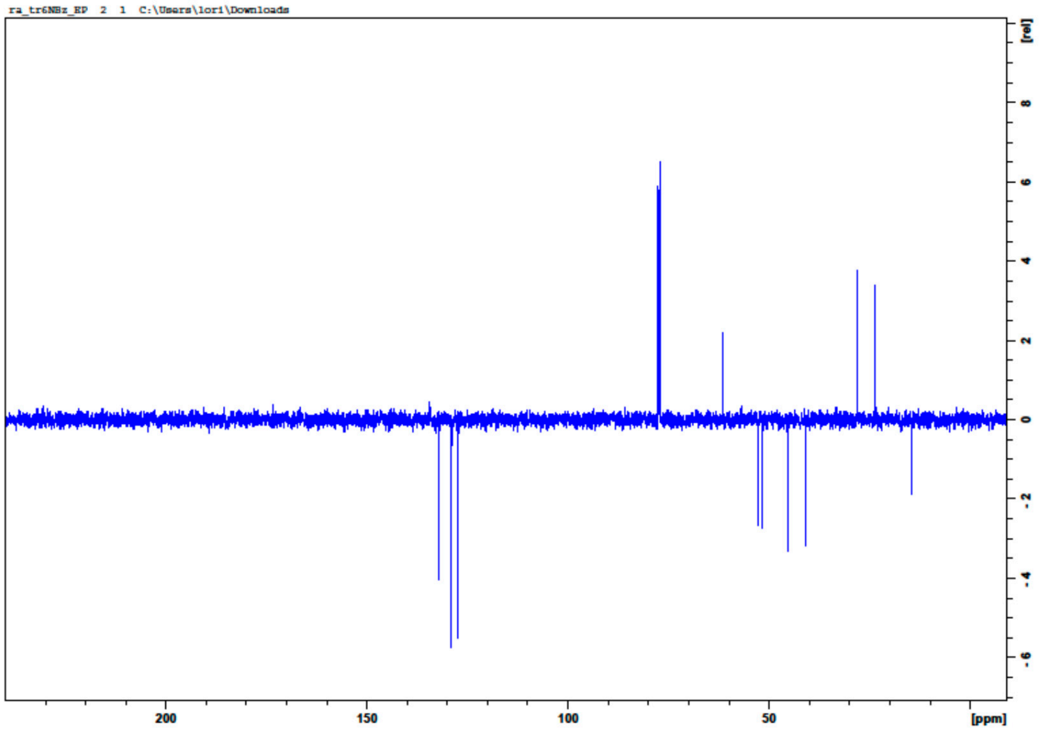

Figure 4. ^{1}H-NMR and ^{13}C-NMR spectra of compound **(±)-13**.

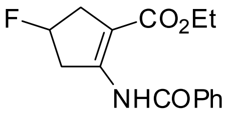

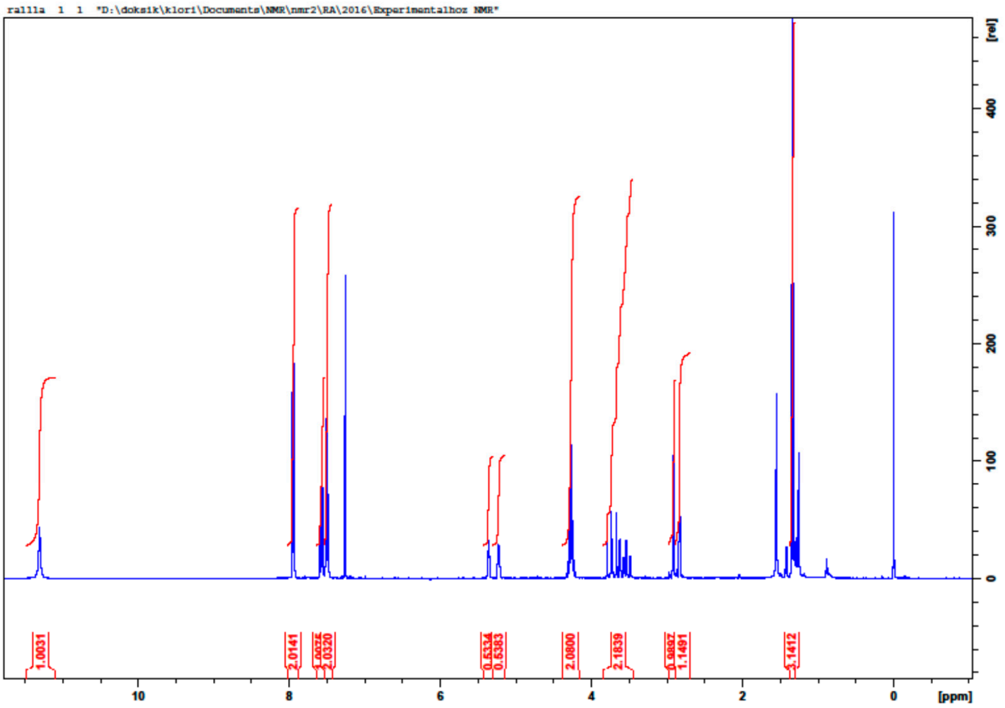

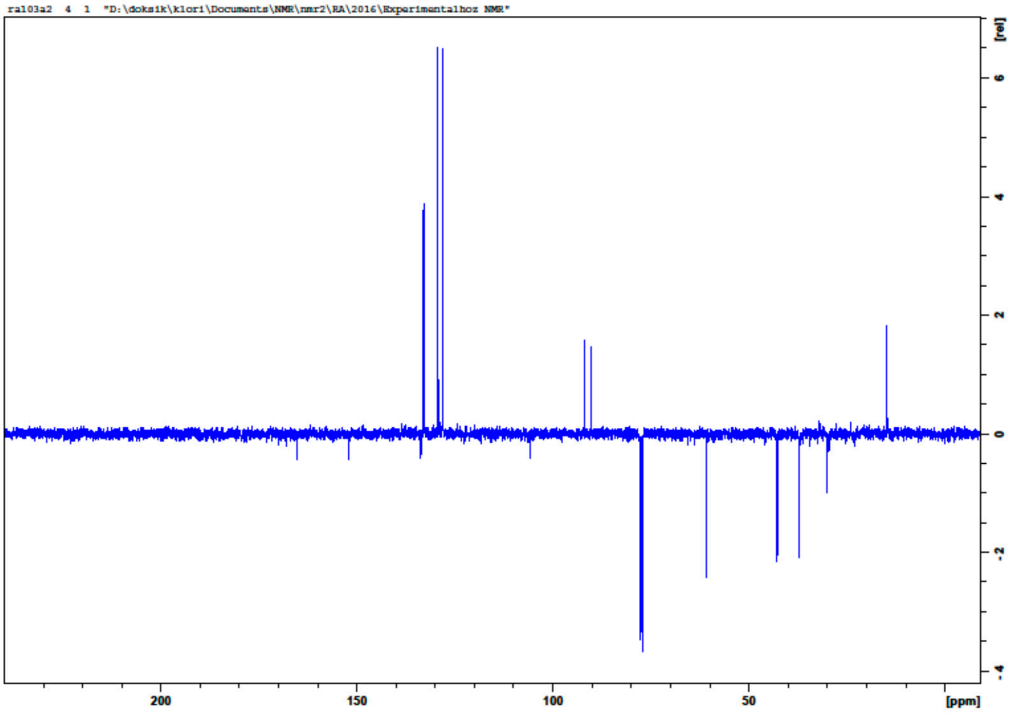

Figure 5. ¹H-NMR and ¹³C-NMR spectra of compound **(±)-3**.

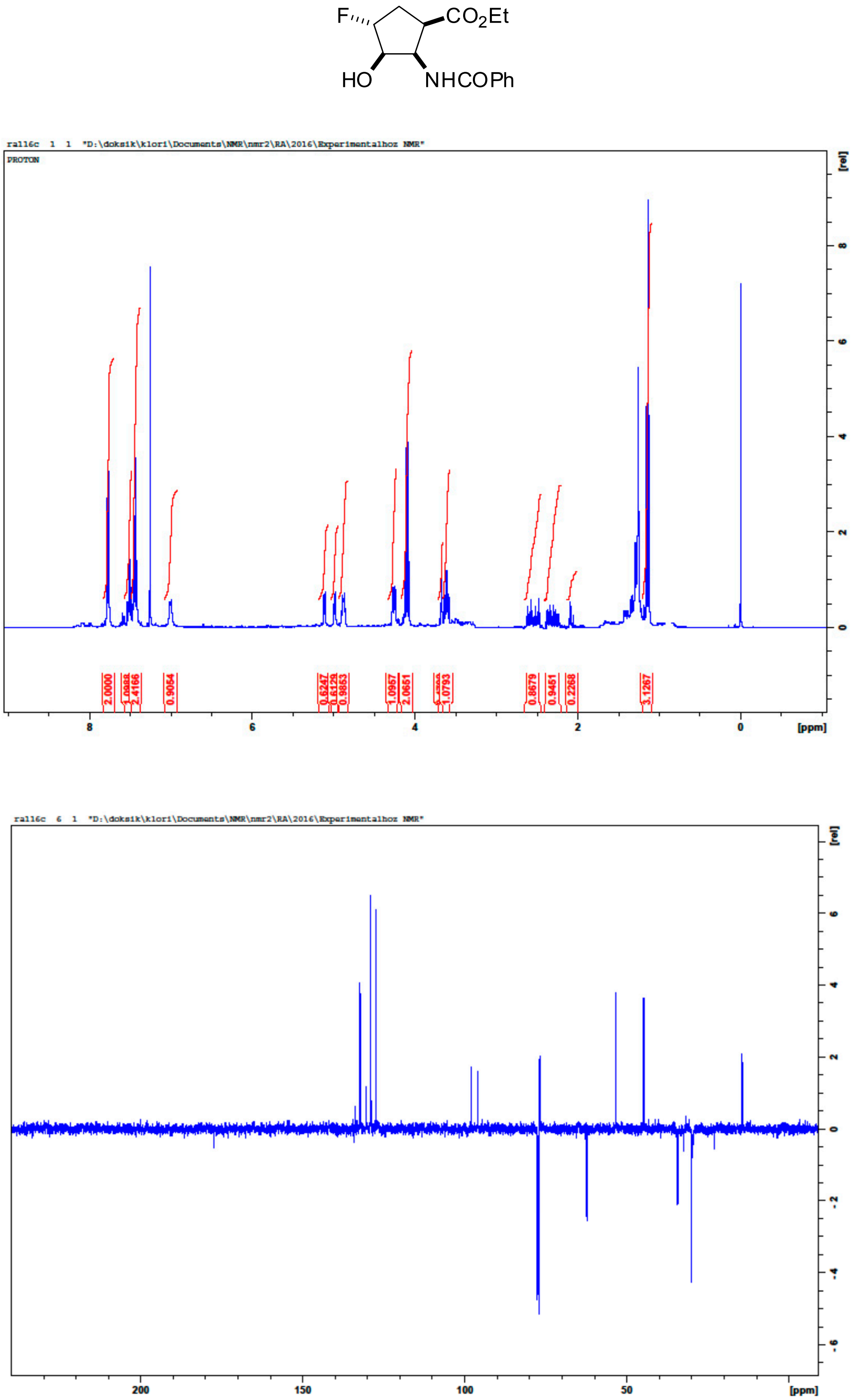

Figure 6. ^{1}H-NMR and ^{13}C-NMR spectra of compound **(±)-4.**

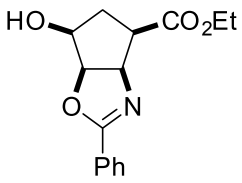

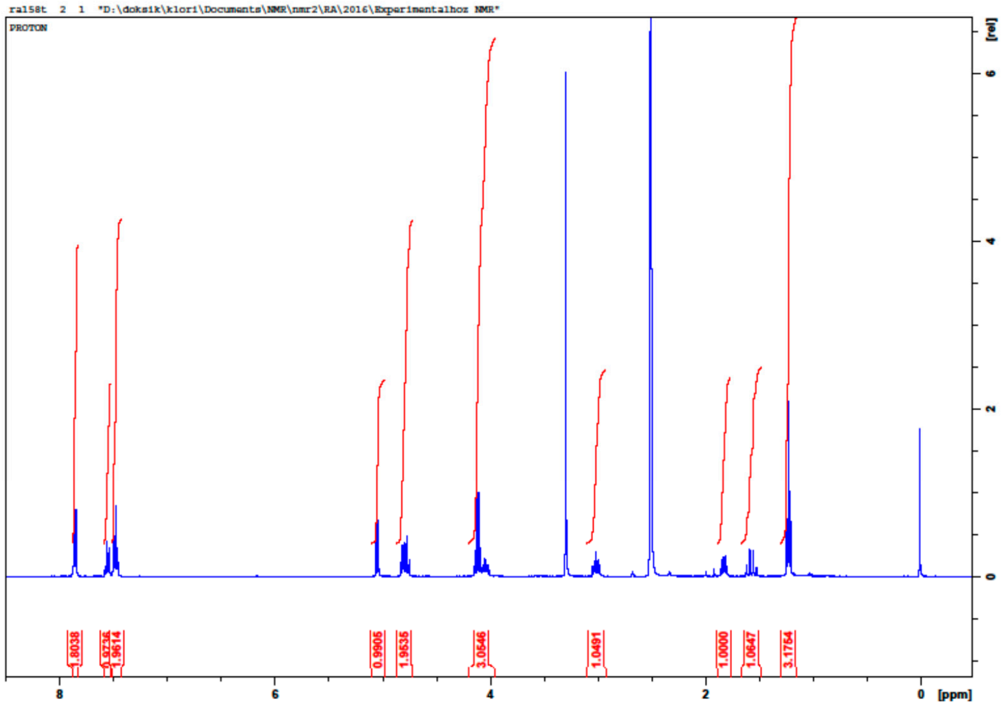

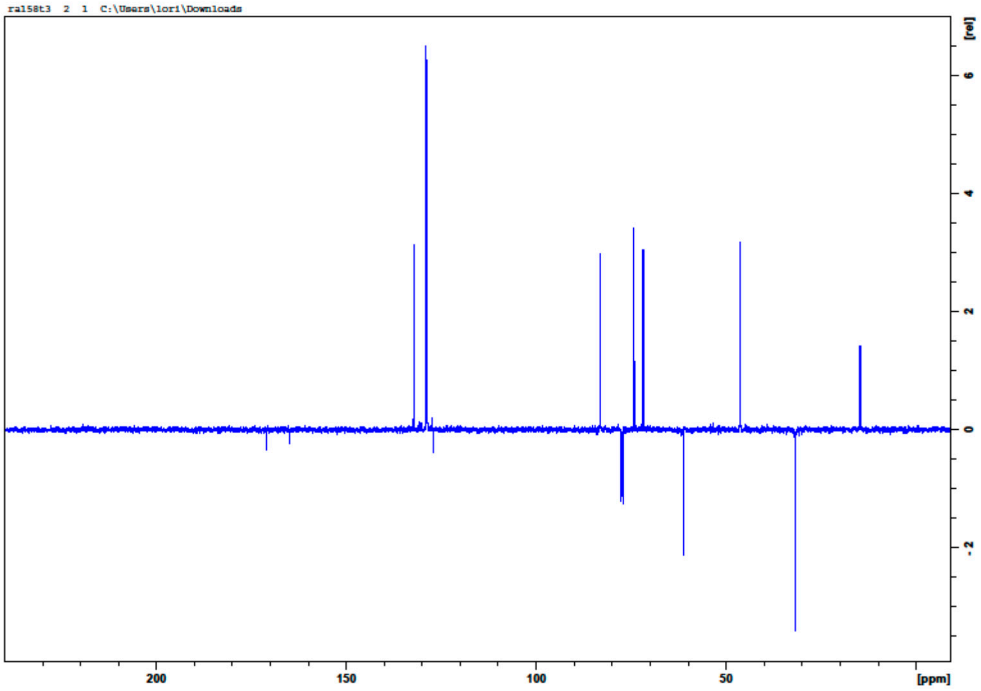

Figure 7. ^{1}H-NMR and ^{13}C-NMR spectra of compound **(±)-5**.

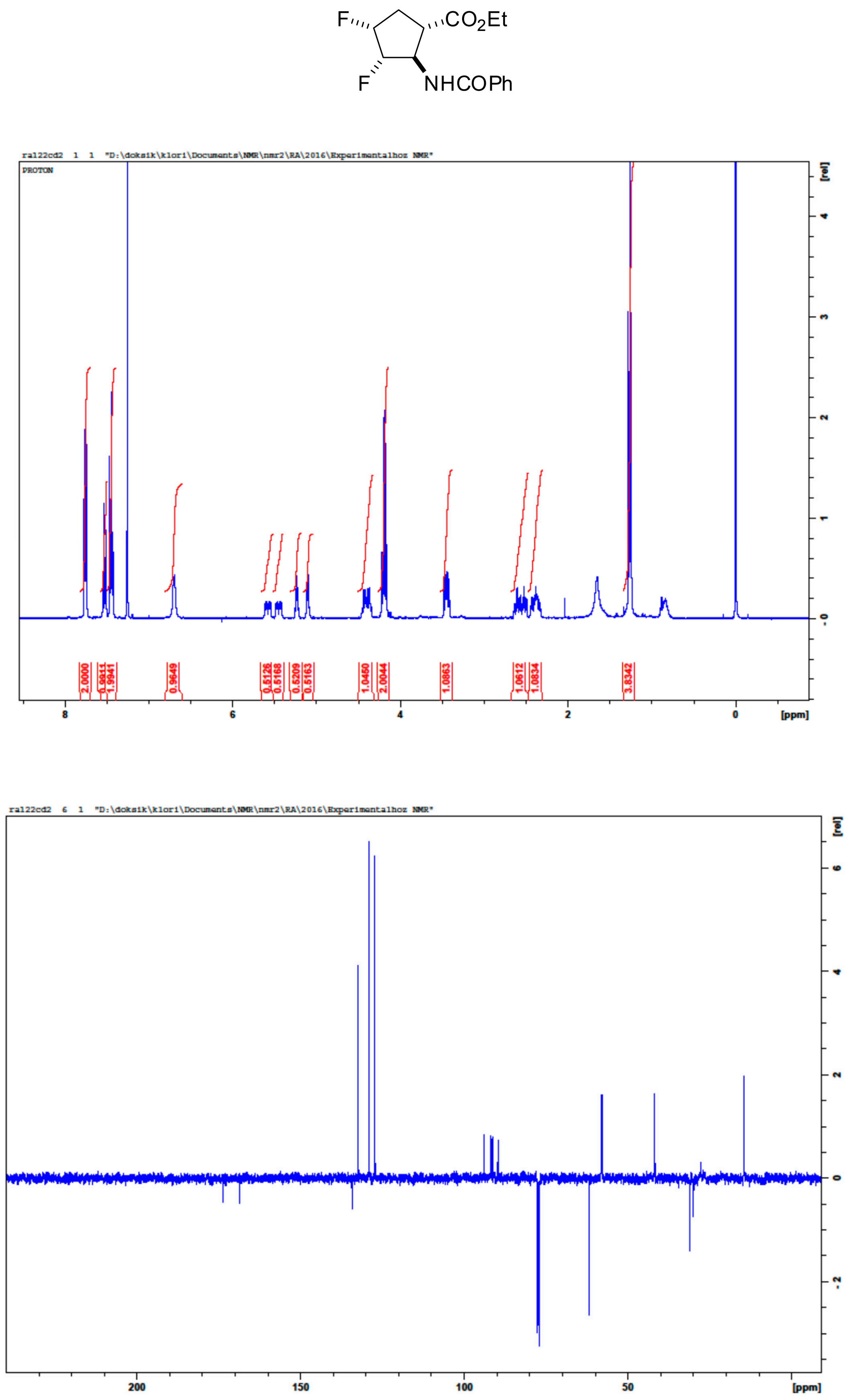

Figure 8. ^{1}H-NMR and ^{13}C-NMR spectra of compound **(±)-7**.

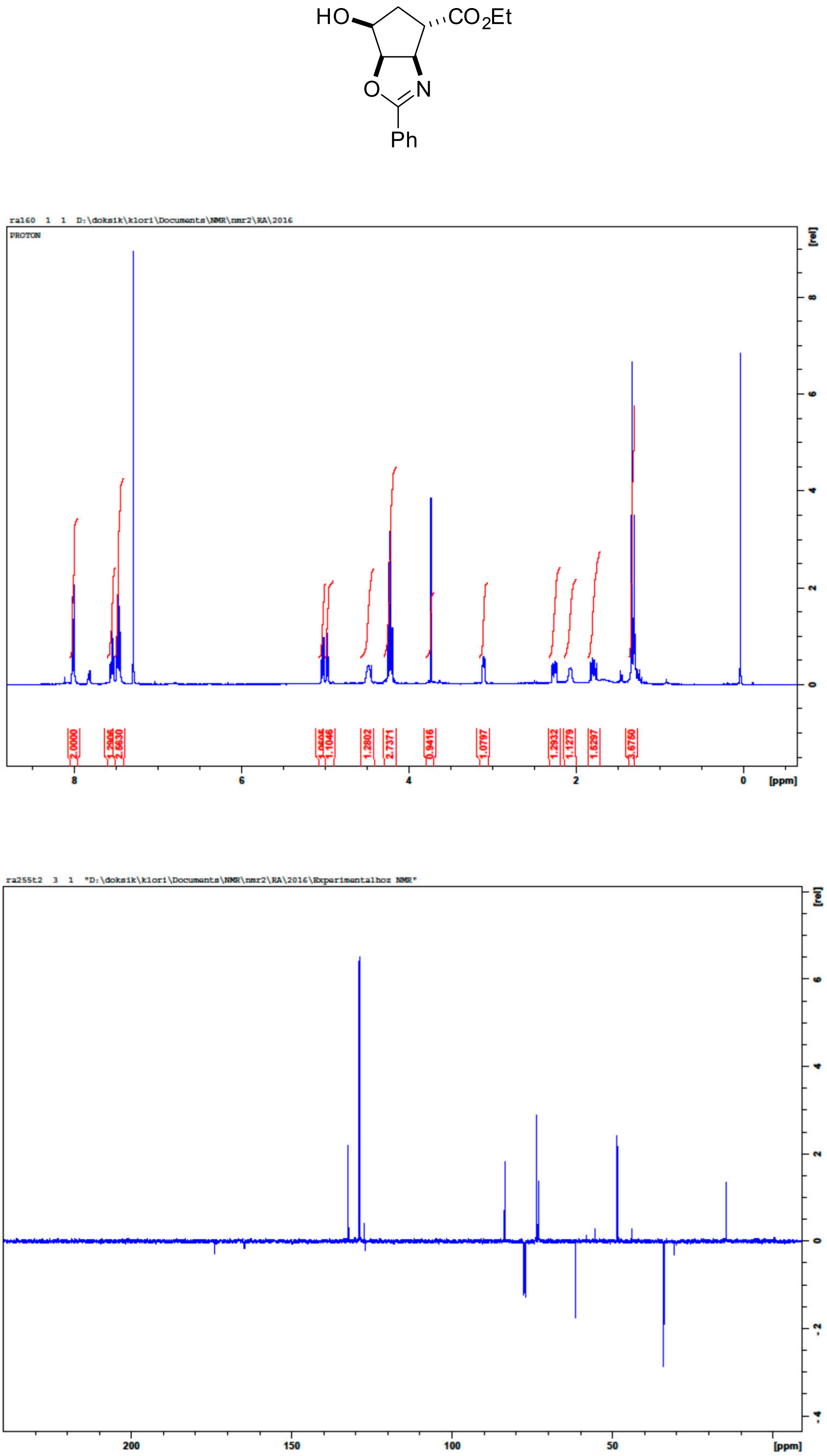

Figure 9. ^{1}H-NMR and ^{13}C-NMR spectra of compound **(±)-8**.

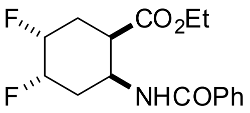

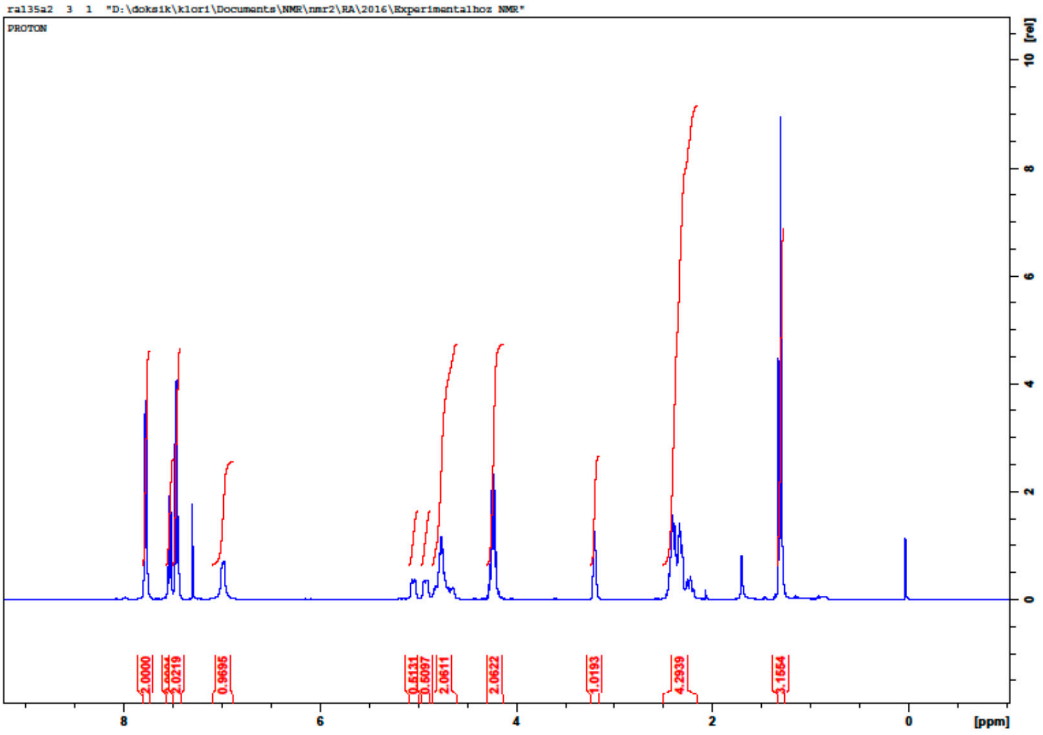

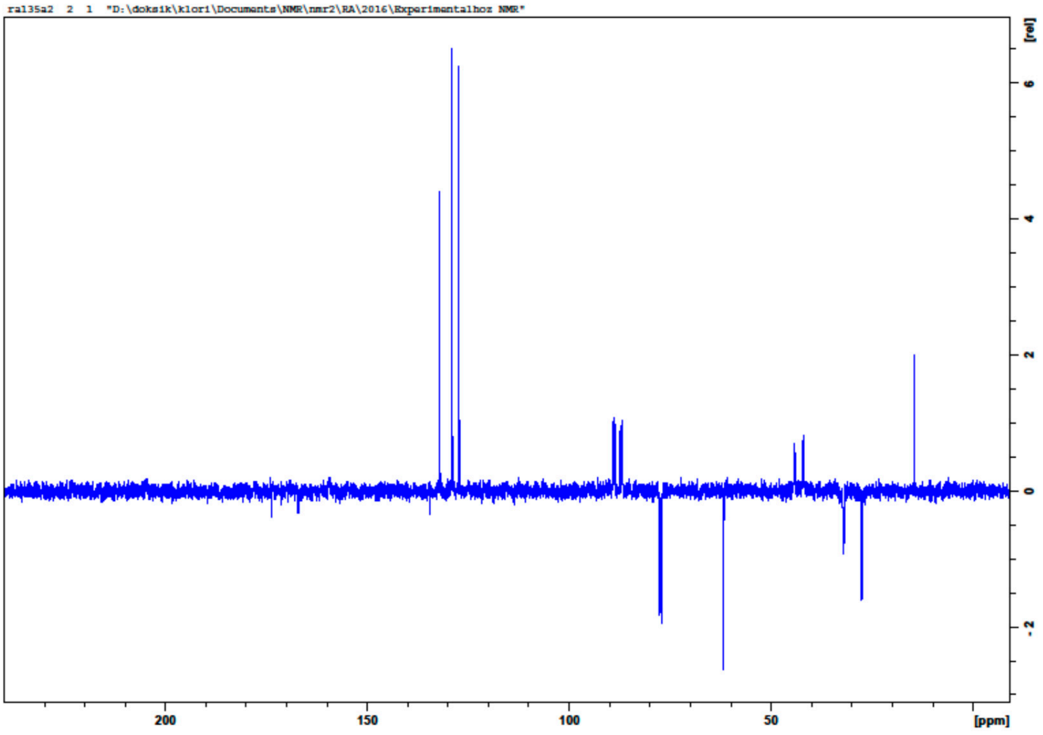

Figure 10. ^{1}H-NMR and ^{13}C-NMR spectra of compound **(±)-11**.

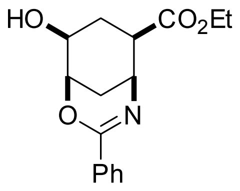

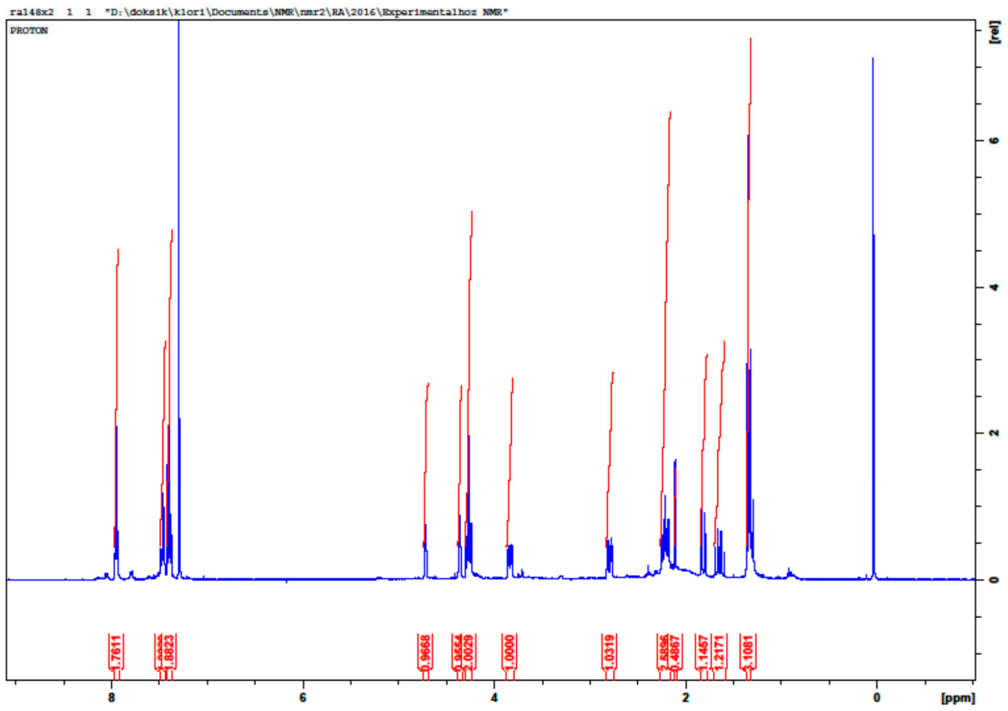

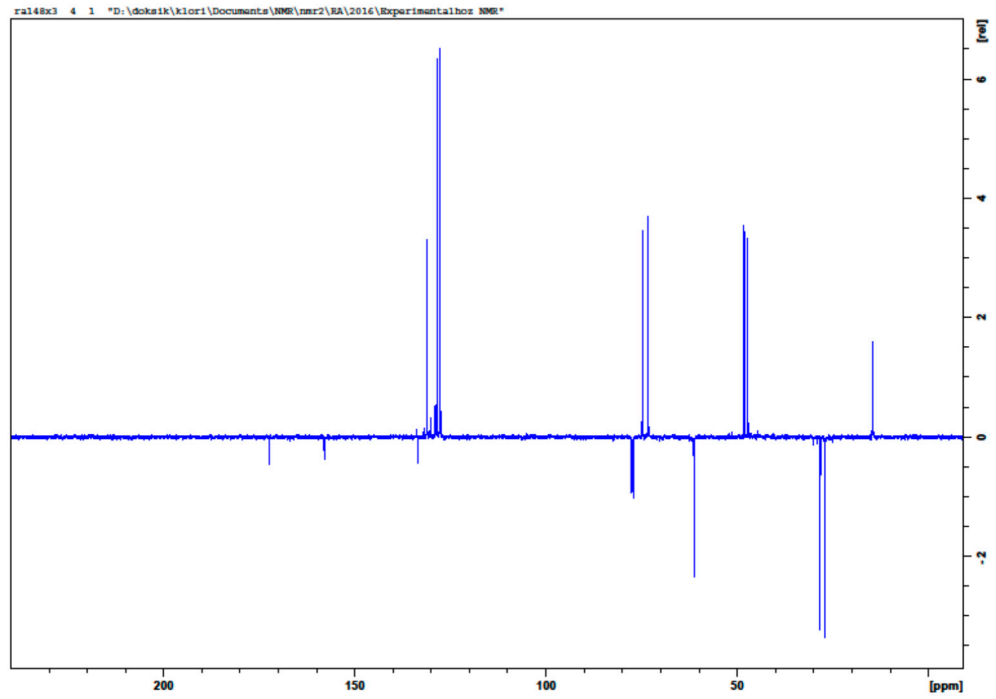

Figure 11. ^{1}H-NMR and ^{13}C-NMR spectra of compound **(±)-12**.

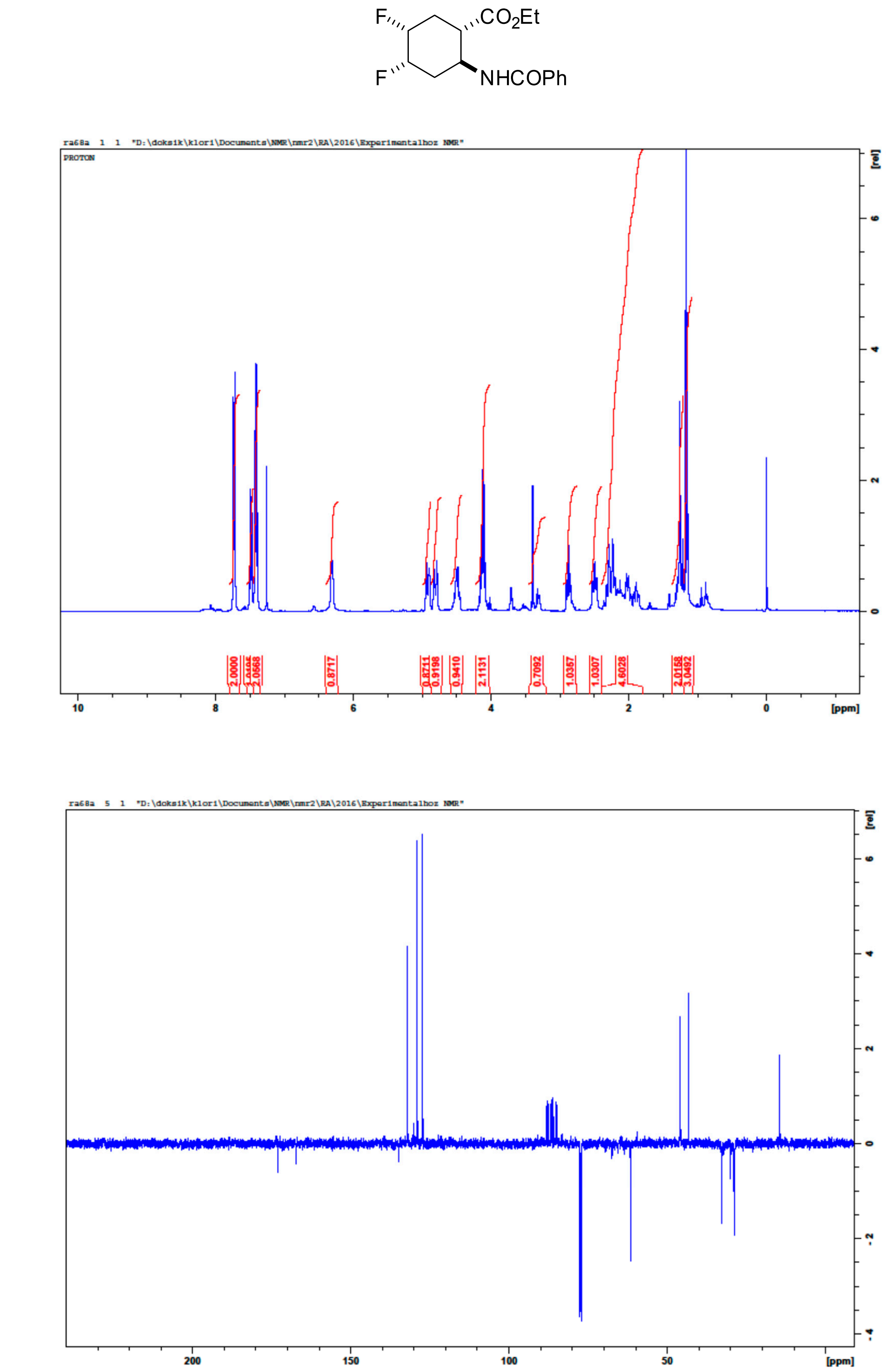

Figure 12. ^{1}H-NMR and ^{13}C-NMR spectra of compound **(±)-15**.

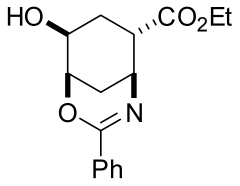

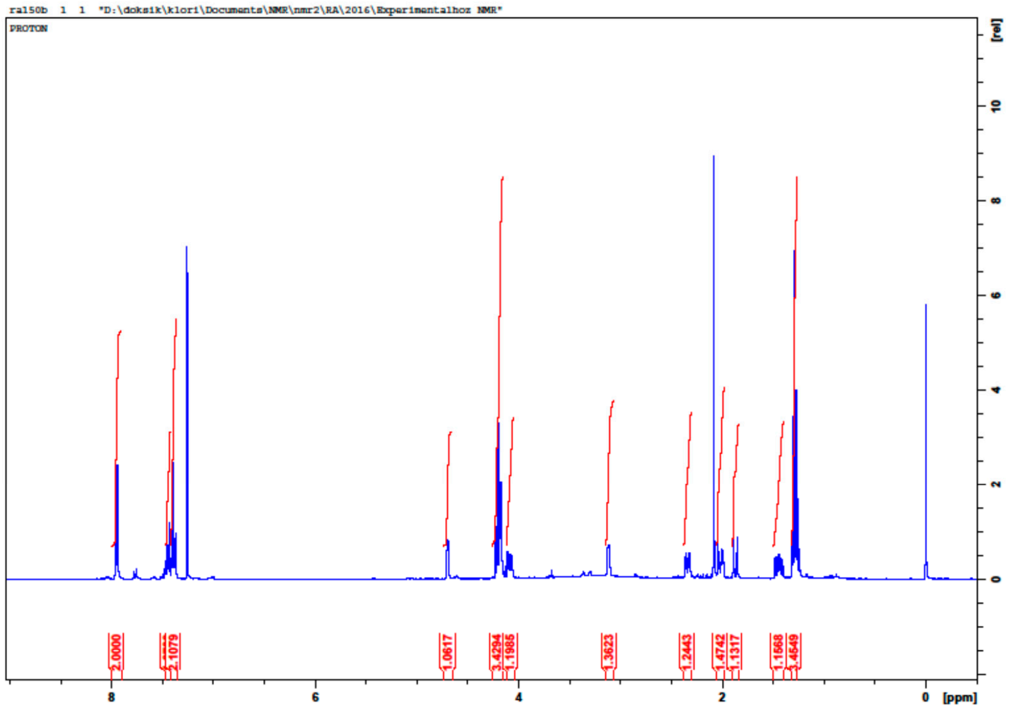

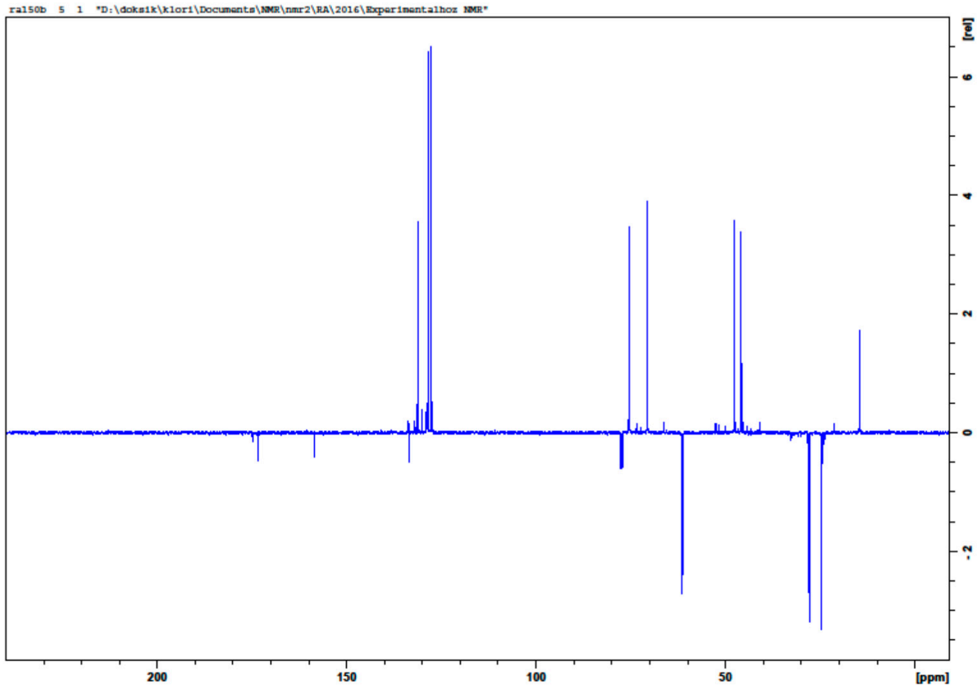

Figure 13. ^{1}H-NMR and ^{13}C-NMR spectra of compound **(±)-14**.

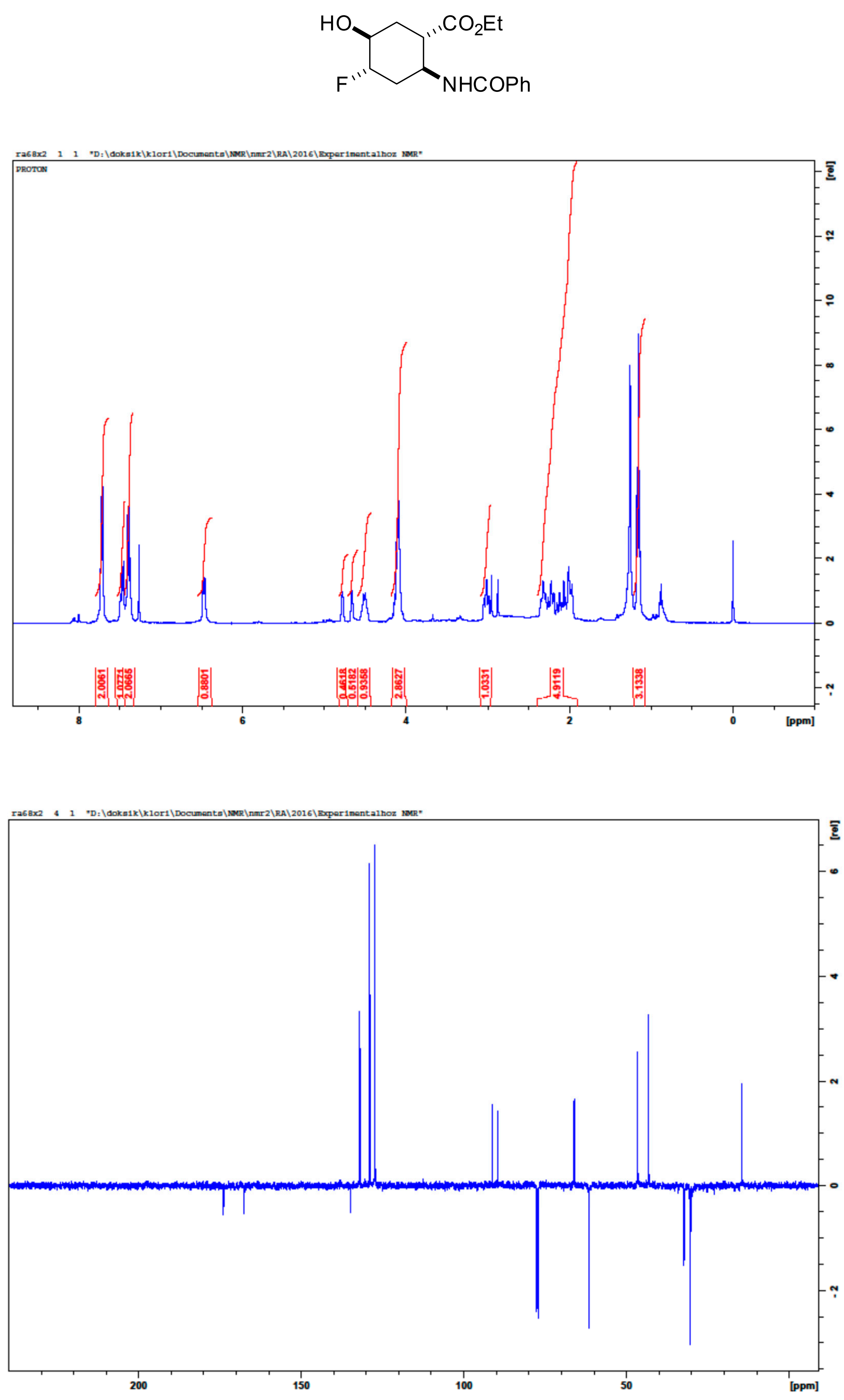

Figure 14. ^{1}H-NMR and ^{13}C-NMR spectra of compound **(±)-16.**
